# Supplementary material for: Spatially organized cellular communities form the developing human heart
Source: Nature. 2024 Mar 13;627(8005):854–64. doi: 10.1038/s41586-024-07171-z (PMC10972757; doi:10.1038/s41586-024-07171-z)
Supplement: Supplementary file 1 — Supplementary Notes 1–4 containing additional details to the text and data presented in the main text, Supplementary Discussion, Supplementary Figs. 1–19 and Supplementary References. [file 41586_2024_7171_MOESM1_ESM.pdf]

nature portfolio

https://doi.org/10.1038/s41586-024-07171-z

Supplementary information

Spatially organized cellular communities form the developing human heart

In the format provided by the authors and unedited

Supplementary Note 1. Single-cell transcriptomic analysis reveals diverse cell lineages constructing the developing human heart.

Although displaying diverse cellular heterogeneity, the cardiomyocyte (CM) compartment segregated into three cell classes which included not only distinct cardiomyocytes from the atrial and ventricular cardiac chambers as described[1–5], but also non-chambered cardiomyocytes (Supplementary Fig. 3). A large proportion of the cardiomyocyte diversity was observed unexpectedly in atrial and ventricular cardiomyocyte classes, which consisted of more specific cell populations that were associated with developmental stages or regions of the heart (Supplementary Fig. 3d, e). In particular, *NR2F2* expressing atrial cardiomyocytes (aCM) partitioned into non-proliferating left and right aCMs, as detected by *PITX2/TRPM3* and *ANGPT1*/*ADM* markers, respectively, as well as *MKI67*+ proliferative cardiomyocytes that were present in both left and right atria but lowly expressed either left or right atrial markers (Supplementary Fig. 3d, e). In contrast to the proliferative aCMs, these left and right aCMs further subdivided into several cell subpopulations that corresponded to specific developmental ages, suggesting their continuing differentiation during heart development (Supplementary Fig. 3d). On the other hand, *MYL2*/*IRX4* expressing ventricular cardiomyocytes (vCM) partitioned into populations correlating to developmental age and the differential expression of maturity-related sarcomeric and metabolic genes such as *LMOD3* and *CKMT2,* which are involved in cardiomyocyte actin filament organization and oxidative phosphorylation[6,7] (Supplementary Fig. 3d, e). Finally, our examination of all regions of the heart combined with our relatively high number of cells sequenced compared to prior cardiac developmental studies[1,5,8–10],

enabled the identification of more rare cardiomyocytes including *BMP2*+ non-chamber cardiomyocytes[11] (ncCM) and an unknown non-descript *TTN*+ cardiomyocyte that may correspond to the recently reported *MYOZ2*-enriched cardiomyocyte[1,12] (Supplementary Fig. 3d, e). These *BMP2*+ non-chambered cardiomyocytes, which have not been as molecularly well-characterized in developing human hearts[1,5,8–10,13], primarily segregated into *RSPO3/MSX2*+ atrioventricular canal/node (ncCM-AVC-like) and *SHOX2/TBX18*+ inflow tract/pacemaker cardiomyocytes (ncCM-IFT-like), which differentially expressed *ISL1, PITX2* and *TBX3,* known transcription factors involved in regulating pacemaker sinoatrial node versus inflow tract development[11,14] (Supplementary Fig. 3d, e). Thus, these findings provide new developmental insight into not only previously reported chamber-related cardiomyocytes[1–5,10,13] but also more specialized cardiomyocytes critical for regulating electrical cardiac conduction.

The mesenchymal compartment exhibited cellular heterogeneity comparable to the cardiomyocyte compartment and divided into three classes: epicardial, fibroblast-like and vascular support cells. In particular, these cells displayed considerable transcriptional similarity with each other (Fig. 1a, Supplementary Fig. 4), suggesting that many of them are developmentally related and may originate from *TBX18+/FRZB+* epicardium-derived progenitor cells (EPDCs) as reported[15]. Consistent with this notion, EPDCs, vascular support cells, and many fibroblast-like cells expressed *TBX18* and *FRZB*, and could be partitioned into *TCF21+/PDGRFA*+ EPDCs and fibroblast-like cells versus *TCF21-/PDGFRB*+ vascular support cells (Supplementary Fig. 4d, e). While vascular support cells were composed of *MYH11*+ vascular smooth muscle cells (VSMC) and *MYH11-/KCNJ8*+ pericytes (4 subpopulations), fibroblast-like cells

exhibited greater cellular complexity (17 subpopulations) and segregated into *DPT*+ fibroblasts and *HAPLN1*+ valvular interstitial cells (VICs). These *DPT*+ fibroblasts classified primarily based on their anatomic location (atrium, *TNC+/MSC+/FDNC1+*; ventricle, *HHIP+/TNC-/MSC-/FDNC1-*) and expression of proliferative markers (*MKI67+/PCNA+),* whereas *HAPLN1*+ VICs further divided into subpopulations correlating to developmental age (Supplementary Fig. 4d, e). In contrast, a subset of *HAPLN1+/PENK*+ valvular interstitial cells (VICs) as well as *OSR1+/TECRL*+ dorsal mesenchymal protrusion (DMP) cells were also discovered that transcriptionally differed from EPDC-related fibroblasts and VICs. These VICs and DMP cells were characterized by their lower expression of *TBX18* and *TCF21* (Supplementary Fig. 4d, e)*,* suggesting that they may derive from alternative sources such as endocardial cells and the second heart field, respectively, as described[16,17]. Supporting their transient developmental role in creating the atrial septum and atrioventricular (AV) cushions[17,18], these DMP cells were observed in the atria and only at 9 and 11 p.c.w. (Supplementary Fig. 4d, e). On the other hand, all VICs were mainly observed in the ventricle but a small proportion of *TBX18*+ EPDC-related VICs could be detected in the atria, suggesting differing roles and contributions between *TBX18*+ versus *TBX18*- VICs[16]. Despite these mesenchymal cells displaying transcriptional overlap and potential functional similarity, our detailed single cell analysis provides new insight into the developmental complexity of these similar cells.

Finally, endothelial, blood and neuronal cell compartments together contributed to ~40% of the observed cardiac cellular heterogeneity but each displayed distinctive cellular diversity (Supplementary Figs. 5-7). *PECAM1*+ endothelial/endocardial cells

grouped into three major classes including *CLDN5+/LEPR-/LYVE-* blood endothelial cells (BEC), *CLDN5+/LEPR-/LYVE1+* lymphatic endothelial cells (LEC) and *CLDN5-/LEPR+/LYVE1-* endocardial cells (Supplementary Fig. 5a, d, e). While BECs subdivided into *HEY1+* arterial, *NR2F2+* venous, *PGF+* capillary and *MKI67+* proliferative endothelial cells, we also observed a spectrum of endocardial cells that in many cases were enriched in either the atrium or ventricle (Supplementary Fig. 5b, d, e). In line with these regional findings, neuronal lineages, which grouped into Schwann and neural crest cells, were primarily observed in the atria (Supplementary Fig. 6b, d, e). In contrast, many known blood cell lineages (white blood cell/WBC and platelet-red blood cell/P-RBC lineages) were discovered throughout all regions of the heart[1–5,8,9,13], but macrophages represented the highest proportion of blood cells, supporting their tissue resident-like role in heart homeostasis as reported[2] (Supplementary Fig. 7).

Supplementary Note 2. Single-cell analyses of the entire developing human heart offer new developmental insights into the diverse cell lineages creating the heart. Our single-cell analyses of the developing human heart have established a comprehensive cell atlas, revealing a diversity of common and rare cell types crucial to the formation of the human heart. Comparative analyses between our developmental human heart cell atlas and a recently published adult human heart cell atlas[19] revealed that several of our identified cell subpopulations match those previously described in adult human hearts including vascular- and neuronal-related cell subpopulations (Supplementary Fig. 8a, b). However, some of our identified cell subpopulations, particularly cardiomyocyte and mesenchymal/fibroblast cells, exhibited diffuse mapping

to adult cell types and thus may be specific for the developing heart (Supplementary Fig. 8a, c, d). For example, developing *BMP2*+ ncCM-IFT-like and ncCM-AVC-like exhibited higher expression of developmental transcription factors (e.g., IFT-like – *SHOX2*, *TBX18*; AVC-like – *TBX3, MSX2*) and lower expression of ion channels (e.g., IFT-like/AVC-like – *CACNA1D, HCN1*) compared to adult pacemaker cardiomyocytes, which also no longer express *BMP2* (Supplementary Fig. 8c). Furthermore, interrogating each dissected cardiac chamber/IVS revealed that many identified cardiac lineages, particularly those inherent to the heart (cardiomyocytes, mesenchymal and endocardial), were region/chamber specific, suggesting that these distinct cardiac lineages become specialized during development to accommodate the function of each cardiac structure (Supplementary Figs. 3-5). To understand how these cardiac cells may develop and specialize in each cardiac region, we performed a cell-cell interaction (CCI) analysis utilizing region specific cells. While we found that each region contained distinct signaling pathways that may regulate the specialization and differentiation of region-specific cardiac cells (Supplementary Fig. 9, Supplementary Table 4), we also discovered growth signaling pathways (e.g., IGF and EDN) present in all regions, which may regulate the proliferation of cardiac cells that contribute to the growth across the heart (Supplementary Fig. 9b, Supplementary Table 4). Consistent with this notion, cardiac cells expressing proliferative markers were frequently observed in all cardiac regions/structures, but expressed lowly cardiac structure-specific genes, suggesting that these cells may be progenitor-like with the potential to expand and then further differentiate into more refined cells within specific cardiac structures (Extended Data Fig. 1, Supplementary Figs. 3-7). In contrast, specialized cardiac cells expressed

combinations of genes specific to each cardiac structure but typically no proliferative markers, and further segregated in many instances according to developmental age, thus supporting that these cells may have differentiated and acquired specific cellular roles based on their regional environment (Supplementary Figs. 3-5). In line with these findings, gene regulatory network (GRN) analyses of these cell populations using pySCENIC (Supplementary Figs. 10, 11) revealed that coordinately regulated gene programs (regulons) positively correlating with developmental aging (i.e., upregulate over time) are related to cell differentiation and function (e.g., muscle contraction for cardiomyocytes, collagen fibril organization for fibroblasts, Supplementary Figs. 10, 11, Supplementary Table 5), whereas regulons negatively correlating with developmental aging (i.e., downregulate over time) are related to general cell processes such as splicing, translation, and cell cycle (Supplementary Figs. 10, 11, Supplementary Table 5). Finally, we performed a developmental trajectory analysis on vCMs, because they exhibit multiple proliferative and developmental cell populations, and identified early stage (early pseudotime) vCMs expressing higher levels of proliferation-related genes (e.g. *H2AFZ*, *TYMS*) but late stage (late pseudotime) vCMs expressing higher levels of sarcomeric genes (e.g. *TTN, MYH7*) (Supplementary Fig. 12a-c, Supplementary Table 6). Applying this trajectory analysis to the GRN and CCI datasets of vCMs enabled the (pseudotime) ordering of GRN factors and cell signaling interactions which may direct the development of vCMs (Supplementary Fig. 12d, e).

Supplementary Note 3. MERFISH-Imputed Spatial Gene Expression

To interrogate how all genes may influence specific cells and their spatial allocation during heart development and morphogenesis, we inferred the full transcriptome for each spatially mapped MERFISH cell by matching similar gene expression profiles between MERFISH cells and cells from corresponding age-matched scRNA-seq data (Fig. 1e). Toward this end, we initially constructed a shared embedding space between cells of these datasets based on the 238 MERFISH target genes using Harmony[20] (Fig. 1e). Nearest neighbor mapping of cells within the joint embedding matched MERFISH cells with similar scRNA-seq cells, thus allowing for the imputation of the expression of missing genes in MERFISH cells using transcriptomic data from corresponding scRNA-seq cells, as well as the transfer of cell annotations between datasets (Fig. 1e). Comparing the cell annotations assigned to the MERFISH cells to those transferred from the scRNA-seq in the joint embedding revealed a high correspondence of analogous cells between MERFISH and scRNA-seq datasets (Fig. 1f), supporting that these datasets are well integrated.

To determine the proficiency of our algorithm to infer the expression of each gene, we analyzed how accurately the transcriptome for any particular cell identified by scRNA-seq or MERFISH could be predicted by extrapolating the scRNA-seq gene expression profiles of its nearest cell neighbor in the shared embedding space (i.e., gene predictability score, Supplementary Fig. 13a, Supplementary Table 15). When predicting the entire transcriptome for these cells, we observed that the scRNA-seq gene predictability scores were low in general (median = 0.024) (Supplementary Fig. 13b). However, when considering only the 3,000 most highly variable genes in the

scRNA-seq dataset, the scRNA-seq gene predictability scores were markedly higher (median = 0.276) (Supplementary Fig. 13b), supporting this algorithm for predicting gene expression relevant to cells of the heart. Confirming that the scRNA-seq gene predictability score can estimate the MERFISH gene predictability score, we discovered that the Pearson correlation between these predictability scores using the 238 MERFISH target genes was 0.71 (Supplementary Fig. 13c). Thus, the scRNA-seq gene predictability scores can be used to determine which other genes can be reliably predicted in MERFISH cells. Supporting this analysis, genes with relatively high scRNA-seq gene predictability scores displayed similar imputed (virtual fluorescent *in situ* hybridization/vFISH) and measured MERFISH spatial expression patterns (Fig. 1g, Supplementary Fig. 13d). To further validate the approach, we re-integrated the scRNA-seq and MERFISH datasets using several quantities of genes (25, 50, 75, 100, 150, and 200 genes) and compared the MERFISH gene predictability score for each quantity (Supplementary Fig. 13e). We discovered that increasing the number of genes used for the integration improved the MERFISH gene predictability scores up to 150 genes, after which the predictability scores plateau (Supplementary Fig. 13e), suggesting that the number of genes used for MERFISH is sufficient to maximize the accuracy achievable by this method to impute the spatial expression of genes not imaged in the MERFISH. Altogether, our integration of MERFISH and scRNA-seq datasets enabled the imputation and spatial mapping of additional genes beyond the examined MERFISH target genes.

Supplementary Note 4. Investigating adult human vCM complexity

To further explore whether adult human vCMs may exhibit cellular complexity similar to vCMs in the developing heart, we compared the transcriptomes of our distinct vCM subpopulations, which were harmonized with our corresponding age-matched scRNA-seq datasets (Supplementary Fig. 15), to those from vCMs discovered in non-failing and diseased adult human ventricles from previous snRNA-seq datasets[21] (Supplementary Fig. 16). Consistent with previous reports that adult human ventricles comprise primarily compact myocardium/vCMs[22,23], we discovered that the majority of non-failing and diseased adult vCMs were compact vCMs as detected by the *RABGAP1L* gene marker, but not necessarily the *HEY2* marker, suggesting that *HEY2* could possibly be a marker for early developing compact vCMs, but *RABGAP1L* may be a marker for more mature adult-like vCMs (Supplementary Fig. 16b). On the other hand, trabecular vCMs were not detected in these human adult vCMs in either non-failing or diseased states using *IRX3* and *GJA5* trabecular vCM markers (Supplementary Fig. 16b). Finally, none of the non-failing nor diseased cardiac gene markers identified in adult cardiomyocytes were expressed in early developing vCM subpopulations (Supplementary Fig. 16c).

Supplementary Discussion

Cardiac multi-modal studies revealed distinct ventricular cell populations interacting and organizing into unexpected multi-layered cellular communities forming the ventricular wall.

The diverse spectrum of cardiovascular lineages that we discovered from our cardiac multi-modal studies included more refined cardiac cell populations, such as cardiomyocytes, cardiac fibroblasts and endocardial/endothelial-related cells, which were oftentimes exclusively identified at specific developmental ages and regions of the heart, supporting that cardiac cell types may adaptively specialize to their environment to accommodate the function of each cardiac structure during development. In particular, we observed an unanticipated wide range of ventricular cardiomyocyte subpopulations that were organized in a complex laminar distribution across the ventricular wall along with other regionally distinct ventricular fibroblast subpopulations. While these ventricular cell subpopulations along with others organized into cellular communities with distinct borders using our community detection algorithm, which assigns each cell to only one community, these cellular communities may be more integrated, exhibiting a more continuous transition between cellular communities across the ventricular walls as observed by the spatial distribution of individual cell populations, particularly cardiomyocytes. Thus, observing both defined communities and the spatial distribution of individual cells provides a more holistic representation of how cardiac cells may interact and organize to create the functional structure of the heart.

PLXN-SEMA interactions direct ventricular wall development and morphogenesis. Our analyses revealed that SEMA3C secreted from compact ventricular fibroblasts may attract *PLXNA2/4*+ trabecular vCMs into the intermediate/compact layer; however, as these migrating trabecular vCMs reach this layer, they encounter endothelial cells expressing membrane-bound SEMA6A and 6B, which may compete with SEMA3C to block/repel the further migration of trabecular vCMs within the intermediate/compact layer (Fig. 5g). Supporting this ventricular wall multi-cellular interaction model and our cardiac PLXN-SEMA results, genetic deletion of Sema3c specifically in cardiac fibroblasts in mice resulted in non-compacted, hypertrabeculated cardiac ventricles. Thus, our combined cardiac PLXN-SEMA findings are consistent with other studies showing that Plexin receptors present on the same cell can differentially respond to various Semaphorins[24–26], and that each interacting Plexin/Semaphorin pair tends to display a gradient of complementary expression, which allows cells to respond to optimal levels of SEMA guidance cues to direct their cellular migration[27,28]. Though the exact mechanisms for how ventricular wall cells expressing the same set of Plexin receptors (PLXNA2/4) respond differentially to these signaling guidance cues and how different levels of receptor/ligand expression affect their cellular response remain to be elucidated, our combined *in vivo* mouse and *in vitro* hPSC studies support that vCMs may respond to a combination of attractive and repulsive signaling cues. However, future *in vivo* and *in vitro* studies may be warranted to fully illuminate how different classes of Plexins and Semaphorins expressed exclusively or combinatorially on distinct cell types may interact to mediate the morphogenesis of the heart and more specifically the ventricular wall.

Supplementary Figures

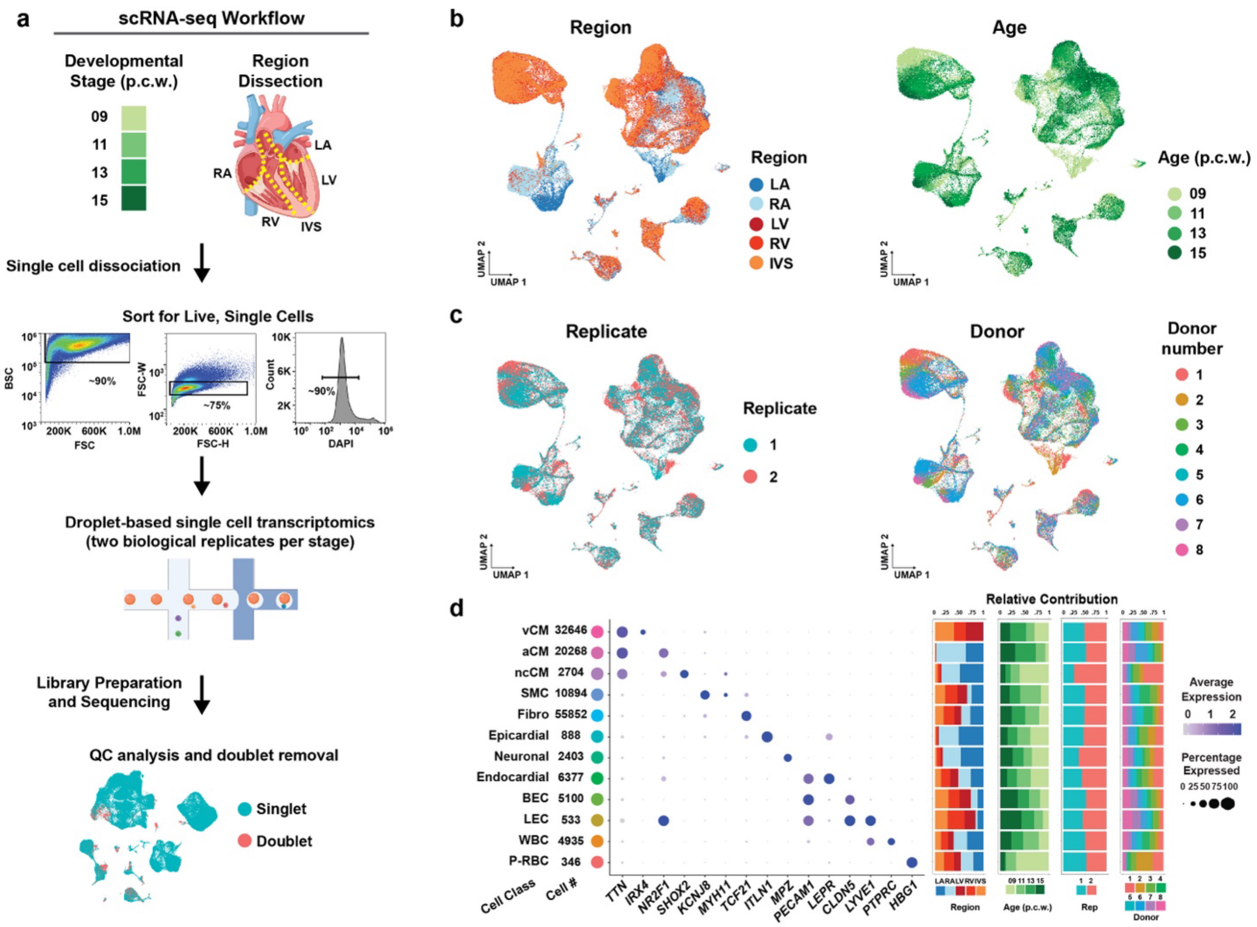

Supplementary Figure 1. Developing human heart scRNA-seq experimental details show the sequencing design and quality control for scRNA-seq datasets.

a, Single-cell RNA sequencing (scRNA-seq) workflow illustrates how samples were collected based on developmental time (9, 11, 13, and 15 p.c.w., two replicates each, eight hearts in total) and region (LA, RA, LV, RV, IVS) and processed for scRNA-seq. **b, c**, Uniform Manifold Approximation and Projection (UMAP) of ~143,000 cardiac cells, colored by (**b**) region and age, and by (**c)** replicate and donor show that scRNA-seq

datasets are well mixed with no appreciable batch effect. **d**, Dot plot of key marker genes identifies each cell class and their distribution across age, region, replicate and donor. aCM, atrial cardiomyocyte; BEC, blood endothelial cell; Fibro, fibroblast; IVS, interventricular septum; LA, left atrium; LEC, lymphatic endothelial cell; LV, left ventricle; ncCM, non-chambered cardiomyocyte; p.c.w., post conception weeks; P-RBC, platelet-red blood cell; RA, right atrium; RV, right ventricle; SMC, smooth muscle cell; vCM, ventricular cardiomyocyte; WBC, white blood cell. Illustration in **a** was created using BioRender (https://www.biorender.com).

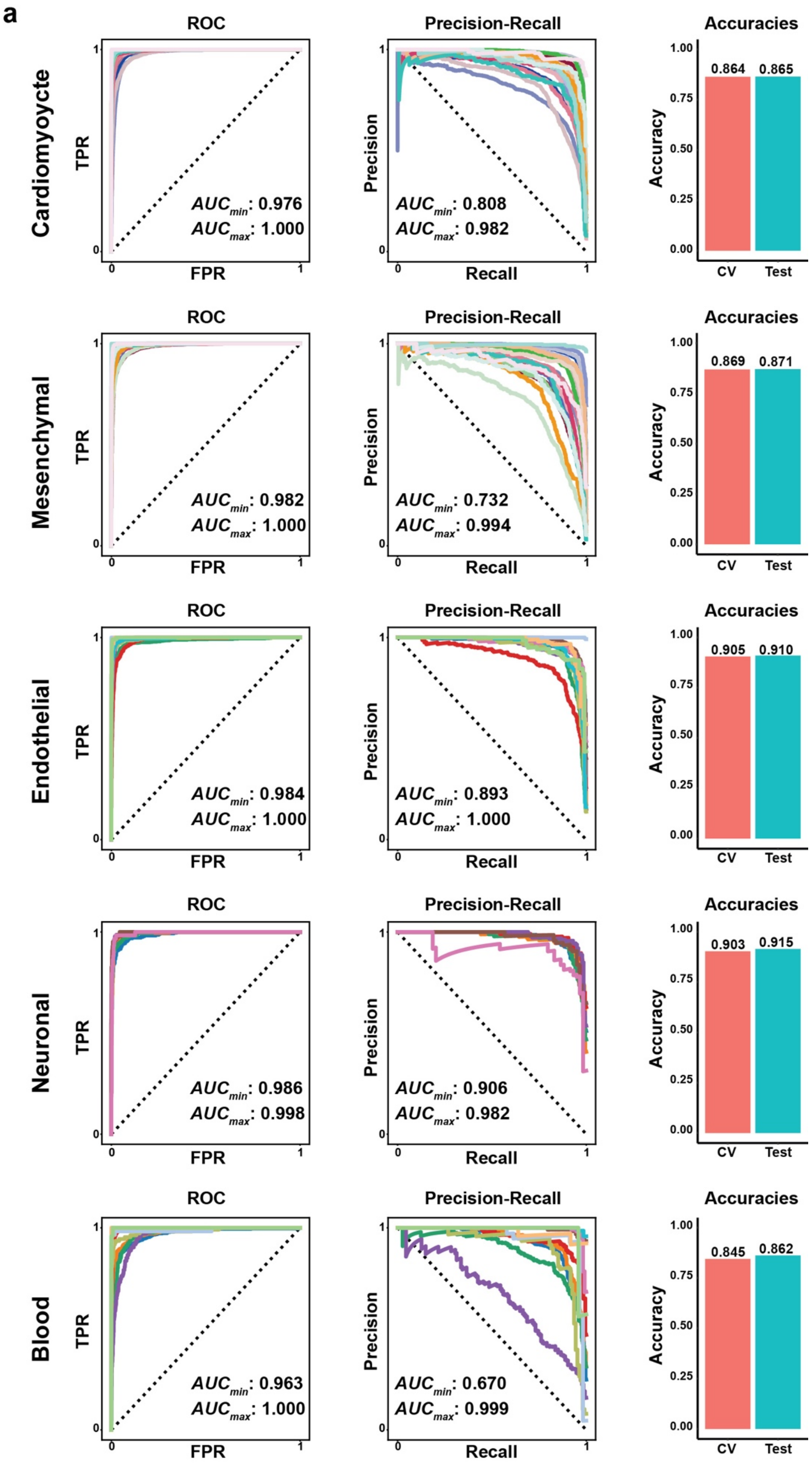
a
Cardiomyocyte
ROC
TPR
FPR
AUCmin: 0.976
AUCmax: 1.000
Precision-Recall
Precision
Recall
AUCmin: 0.808
AUCmax: 0.982
Accuracies
Accuracy
0.864
0.865
CV
Test
Mesenchymal
AUCmin: 0.982
AUCmax: 1.000
AUCmin: 0.732
AUCmax: 0.994
0.869
0.871
Endothelial
AUCmin: 0.984
AUCmax: 1.000
AUCmin: 0.893
AUCmax: 1.000
0.905
0.910
Neuronal
AUCmin: 0.986
AUCmax: 0.998
AUCmin: 0.906
AUCmax: 0.982
0.903
0.915
Blood
AUCmin: 0.963
AUCmax: 1.000
AUCmin: 0.670
AUCmax: 0.999
0.845
0.862

Supplementary Figure 2. SCCAF was used to assess the accuracy for cardiac cell clusters identified in the scRNA-seq datasets. a, Using the SCCAF algorithm, we plotted a receiver operating characteristic (ROC) curve (left plot), a precision-recall curve (middle plot), and accuracies (right plot) for each cell compartment. As a result, the calculated SCCAF accuracies (i.e. test and CV) for each major cell compartment clustering solution were ~85% or greater, and were within 2% of each other. AUC, area under the curve; CV, cross-validation; FPR, false positive rate; TPR, true positive rate.

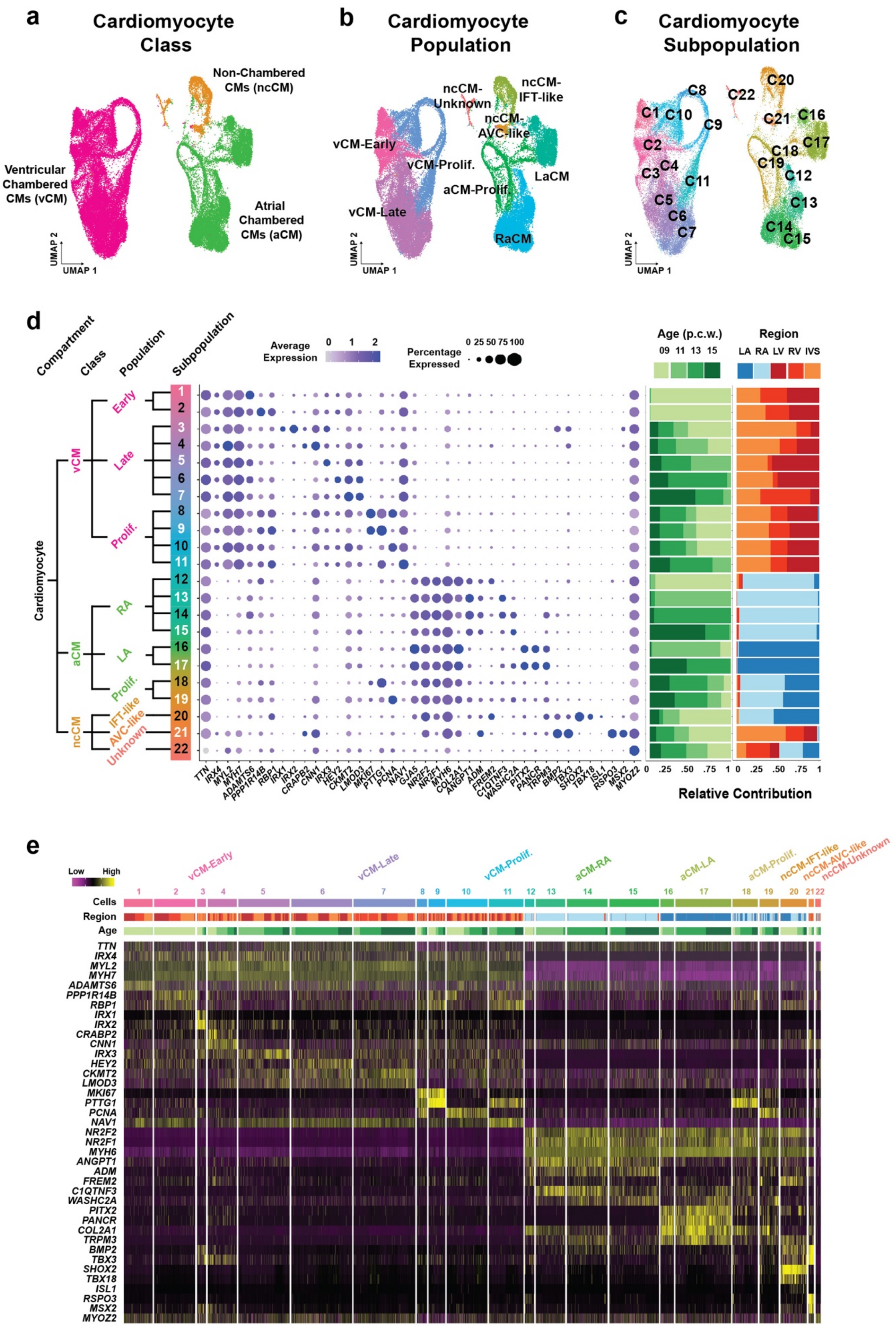
a
Cardiomyocyte Class
Non-Chambered CMs (ncCM)
Ventricular Chambered CMs (vCM)
Atrial Chambered CMs (aCM)
UMAP 2
UMAP 1
b
Cardiomyocyte Population
ncCM-Unknown
ncCM-IFT-like
ncCM-AVC-like
vCM-Early
vCM-Prolif.
aCM-Prolif.
LaCM
vCM-Late
RaCM
UMAP 2
UMAP 1
c
Cardiomyocyte Subpopulation
C1 C2 C3 C4 C5 C6 C7 C8 C9 C10 C11 C12 C13 C14 C15 C16 C17 C18 C19 C20 C21 C22
UMAP 2
UMAP 1
d
Compartment
Class
Population
Subpopulation
Average Expression
0 1 2
Percentage Expressed
0 25 50 75 100
Age (p.c.w.)
09 11 13 15
Region
LA RA LV RV IVS
Cardiomyocyte
vCM
aCM
ncCM
Early
Late
Prolif.
RA
LA
Prolif.
IFT-like
AVC-like
Unknown
1 2 3 4 5 6 7 8 9 10 11 12 13 14 15 16 17 18 19 20 21 22
TTN IRX4 MYL2 MYH7 ADAMTS6 PPP1R14B RBP1 IRX1 IRX2 CRABP2 CNN1 IRX3 HEY2 CKMT2 LMOD3 MKI67 PTTG1 PCNA NAV1 GJA5 NR2F2 NR2F1 MYH6 COL2A1 ANGPT1 ADM FREM2 C1QTNF3 WASHC2A PITX2 PANCR TRPM3 BMP2 TBX3 SHOX2 TBX18 ISL1 RSPO3 MSX2 MYOZ2
0 .25 .50 .75 1
0 .25 .50 .75 1
Relative Contribution
e
Low High
vCM-Early
vCM-Late
vCM-Prolif.
aCM-RA
aCM-LA
aCM-Prolif.
ncCM-IFT-like
ncCM-AVC-like
ncCM-Unknown
1 2 3 4 5 6 7 8 9 10 11 12 13 14 15 16 17 18 19 20 21 22
Cells
Region
Age
TTN
IRX4
MYL2
MYH7
ADAMTS6
PPP1R14B
RBP1
IRX1
IRX2
CRABP2
CNN1
IRX3
HEY2
CKMT2
LMOD3
MKI67
PTTG1
PCNA
NAV1
NR2F2
NR2F1
MYH6
ANGPT1
ADM
FREM2
C1QTNF3
WASHC2A
PITX2
PANCR
COL2A1
TRPM3
BMP2
TBX3
SHOX2
TBX18
ISL1
RSPO3
MSX2
MYOZ2

Supplementary Figure 3. Single-cell transcriptomic profiling reveals three cardiomyocyte classes: atrial-chamber, ventricular-chamber and specialized non-chambered cardiomyocyte. **a**, The cardiomyocyte compartment comprises three major cardiomyocyte classes as displayed in UMAP. These cardiomyocytes were further subdivided into specific cardiomyocyte **b**, populations and **c**, subpopulations. **d**, Dot plot of key marker genes identifies each distinct cardiomyocyte and their distribution across age and region – 3 classes, 9 populations, and 22 subpopulations. **e**, Heatmap shows differentially expressed genes identified for each distinct cardiomyocyte ordered by cell identity, region and age along the x-axis. aCM, atrial cardiomyocyte; AVC, atrioventricular canal; IFT, inflow tract; IVS, interventricular septum; LA, left atrium; LV, left ventricle; ncCM, non-chambered cardiomyocyte; p.c.w., post conception weeks; Prolif., proliferating; RA, right atrium; RV, right ventricle; vCM, ventricular cardiomyocyte.

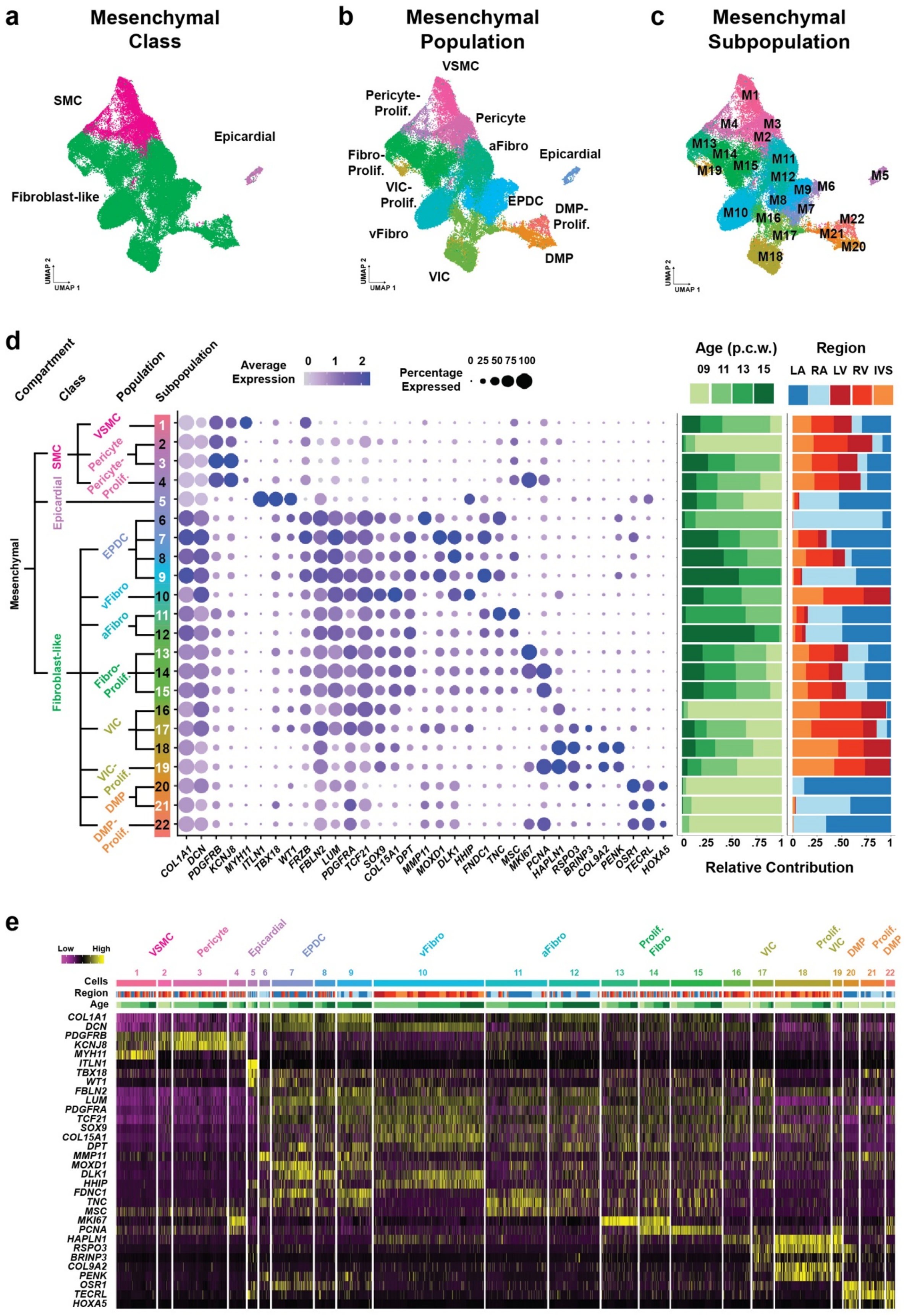
a
Mesenchymal Class
SMC
Epicardial
Fibroblast-like
b
Mesenchymal Population
VSMC
Pericyte-Prolif.
Pericyte
aFibro
Epicardial
Fibro-Prolif.
VIC-Prolif.
EPDC
DMP-Prolif.
vFibro
DMP
VIC
c
Mesenchymal Subpopulation
d
Compartment
Class
Population
Subpopulation
Average Expression
Percentage Expressed
Age (p.c.w.)
09 11 13 15
Region
LA RA LV RV IVS
Mesenchymal
SMC
Epicardial
Fibroblast-like
Relative Contribution
e
Low High
Cells
Region
Age

Supplementary Figure 4. Human heart mesenchymal compartment exhibits relative transcriptional similarity across epicardial, vascular support and fibroblast-like classes. **a**, The mesenchymal compartment comprises three cell classes as displayed in UMAP. Each mesenchymal class was further subdivided into specific cell **b**, populations and **c**, subpopulations. **d**, Dot plot of key marker genes identifies each distinct mesenchymal-related cell and their distribution across age and region – 3 classes, 11 populations, and 22 subpopulations. **e**, Heatmap shows differentially expressed genes identified for each distinct mesenchymal-related cell ordered by cell identity, region and age along the x-axis. aFibro, atrial fibroblast; DMP, dorsal mesenchymal protrusion; EPDC, epicardial-derived cell; IVS, interventricular septum; LA, left atrium; LV, left ventricle; p.c.w., post conception weeks; Prolif., proliferating; RA, right atrium; RV, right ventricle; SMC, smooth muscle cell; vFibro, ventricular fibroblast; VIC, valve interstitial cell; VSMC, vascular smooth muscle cell.

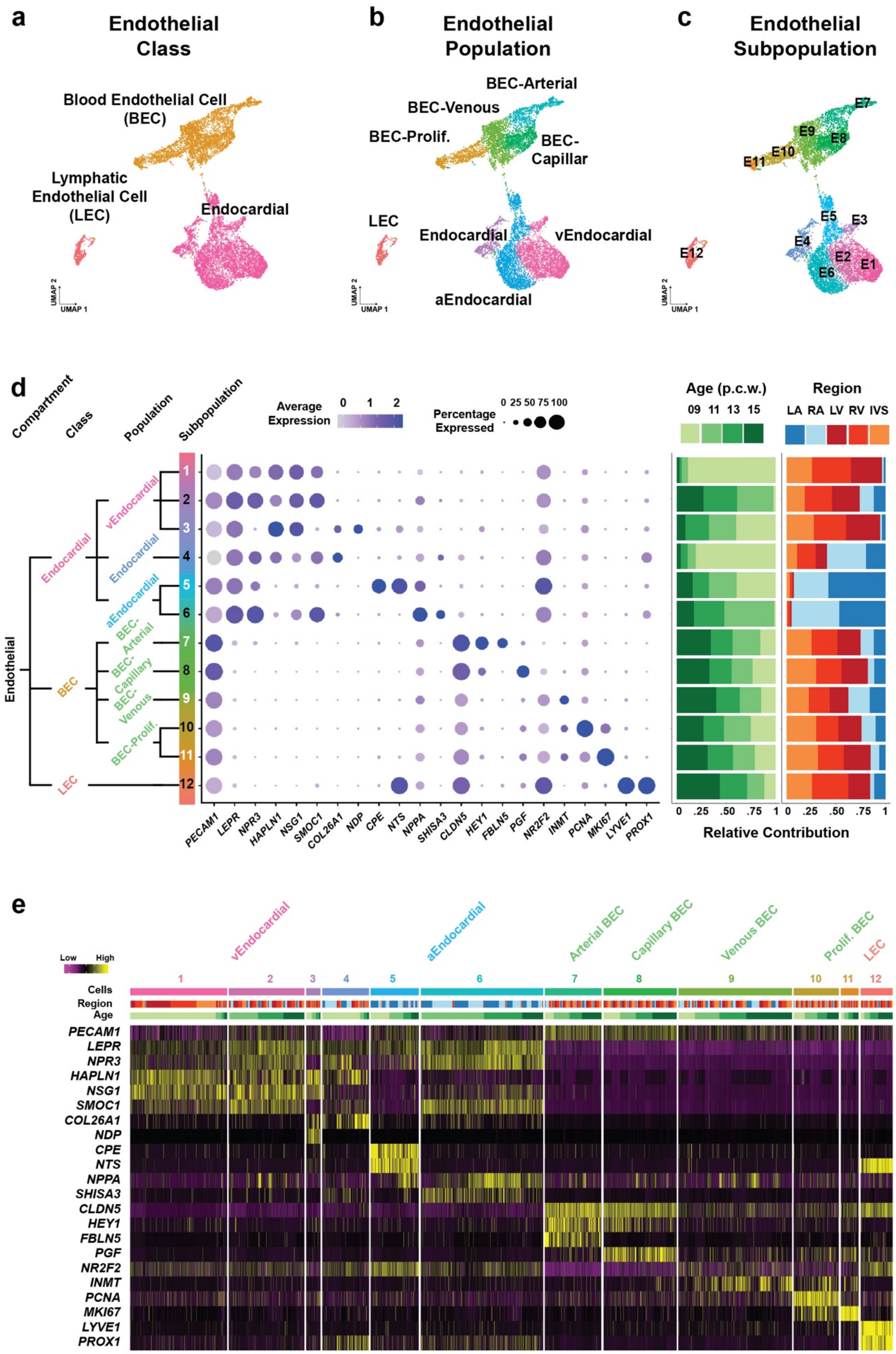
a
Endothelial Class
Blood Endothelial Cell (BEC)
Lymphatic Endothelial Cell (LEC)
Endocardial
UMAP 2
UMAP 1
b
Endothelial Population
BEC-Arterial
BEC-Venous
BEC-Prolif.
BEC-Capillar
LEC
Endocardial
vEndocardial
aEndocardial
c
Endothelial Subpopulation
E7
E9
E8
E10
E11
E5
E3
E4
E2
E1
E6
E12
d
Compartment
Class
Population
Subpopulation
Average Expression
0 1 2
Percentage Expressed
0 25 50 75 100
Endothelial
Endocardial
BEC
LEC
vEndocardial
Endocardial
aEndocardial
BEC-Arterial
BEC-Capillary
BEC-Venous
BEC-Prolif.
1
2
3
4
5
6
7
8
9
10
11
12
PECAM1
LEPR
NPR3
HAPLN1
NSG1
SMOC1
COL26A1
NDP
CPE
NTS
NPPA
SHISA3
CLDN5
HEY1
FBLN5
PGF
NR2F2
INMT
PCNA
MKI67
LYVE1
PROX1
Age (p.c.w.)
09 11 13 15
Region
LA RA LV RV IVS
0 .25 .50 .75 1
0 .25 .50 .75 1
Relative Contribution
e
Low
High
vEndocardial
aEndocardial
Arterial BEC
Capillary BEC
Venous BEC
Prolif. BEC
LEC
1
2
3
4
5
6
7
8
9
10
11
12
Cells
Region
Age
PECAM1
LEPR
NPR3
HAPLN1
NSG1
SMOC1
COL26A1
NDP
CPE
NTS
NPPA
SHISA3
CLDN5
HEY1
FBLN5
PGF
NR2F2
INMT
PCNA
MKI67
LYVE1
PROX1

Supplementary Figure 5. Three endothelial classes including blood endothelial cell (BEC), lymphatic endothelial cell (LEC) and endocardial cell were identified in the human heart. **a**, The endothelial compartment comprises three classes as displayed in UMAP. Each endothelial class was further subdivided into specific endothelial-like cell **b**, populations and **c**, subpopulations. **d**, Dot plot of key marker genes identifies each distinct endothelial-related cell and their distribution across age and region – 3 classes, 8 populations, and 12 subpopulations. **e**, Heatmap shows differentially expressed genes discovered for each identified endothelial cell ordered by cell identity, region and age along the x-axis. aEndocardial, atrial endocardial; BEC, blood endothelial cell; IVS, interventricular septum; LA, left atrium; LEC, lymphatic endothelial cell; LV, left ventricle; p.c.w., post conception weeks; Prolif., proliferating; RA, right atrium; RV, right ventricle; vEndocardial, ventricular endocardial.

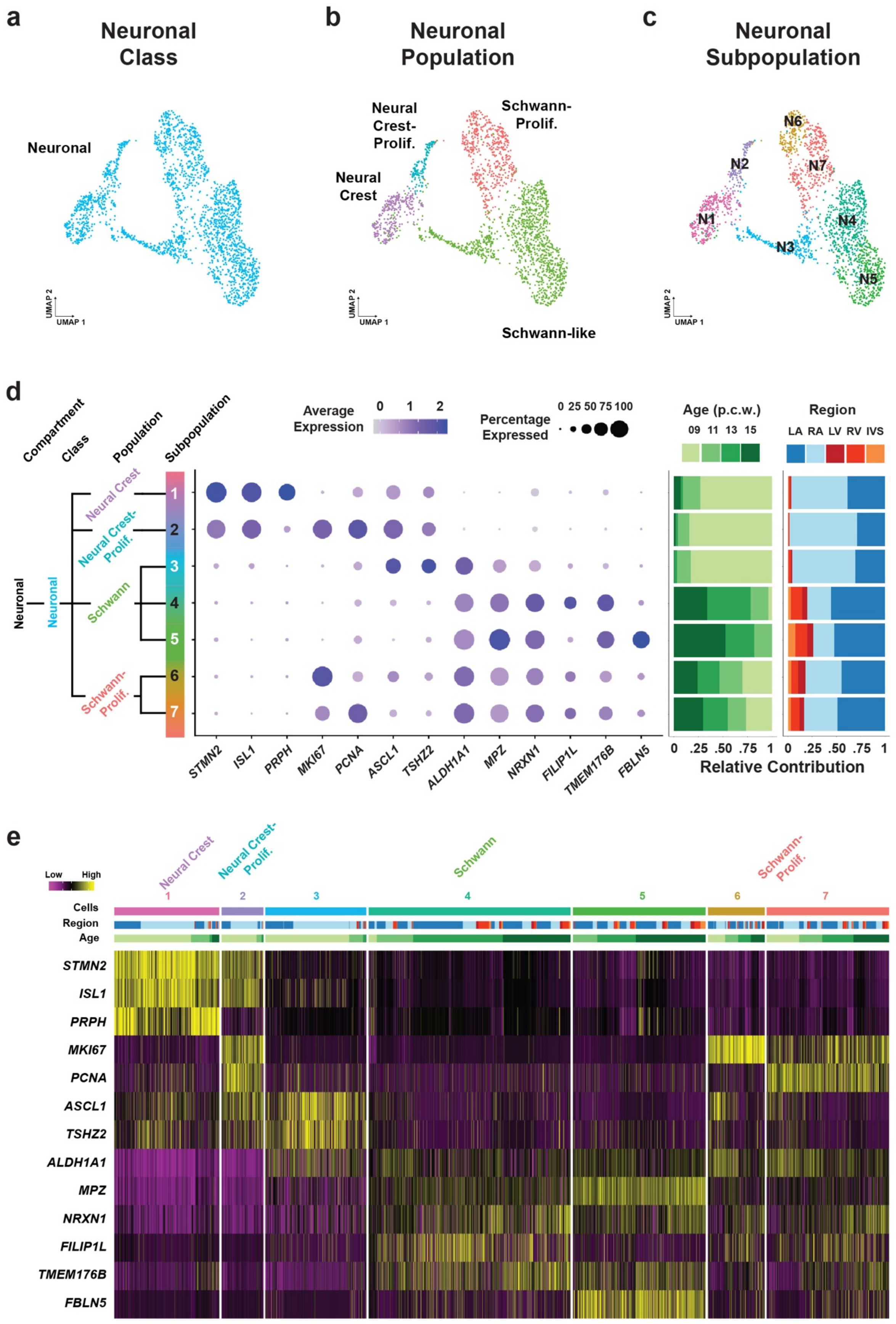
a
Neuronal Class
Neuronal
UMAP 2
UMAP 1
b
Neuronal Population
Neural Crest-Prolif.
Schwann-Prolif.
Neural Crest
Schwann-like
c
Neuronal Subpopulation
N1
N2
N3
N4
N5
N6
N7
d
Compartment
Class
Population
Subpopulation
Average Expression
Percentage Expressed
Age (p.c.w.)
09 11 13 15
Region
LA RA LV RV IVS
Neuronal
Neural Crest
Neural Crest-Prolif.
Schwann
Schwann-Prolif.
STMN2
ISL1
PRPH
MKI67
PCNA
ASCL1
TSHZ2
ALDH1A1
MPZ
NRXN1
FILIP1L
TMEM176B
FBLN5
Relative Contribution
e
Low
High
Cells
Region
Age

Supplementary Figure 6. The neuronal compartment of the human heart is enriched in the atria and composed of neural crest and Schwann cells. a, The neuronal compartment contains one major cell class as displayed in UMAP, which was further segregated into specific neuronal **b**, populations and **c**, subpopulations. **d**, Dot plot of key marker genes identifies each distinct neuronal cell and their distribution across age and region – 1 class, 4 populations, and 7 subpopulations. **e**, Heatmap shows differentially expressed genes identified for each distinct neuronal cell ordered by cell identity, region and age along the x-axis. IVS, interventricular septum; LA, left atrium; LV, left ventricle; p.c.w., post conception weeks; Prolif., proliferating; RA, right atrium; RV, right ventricle.

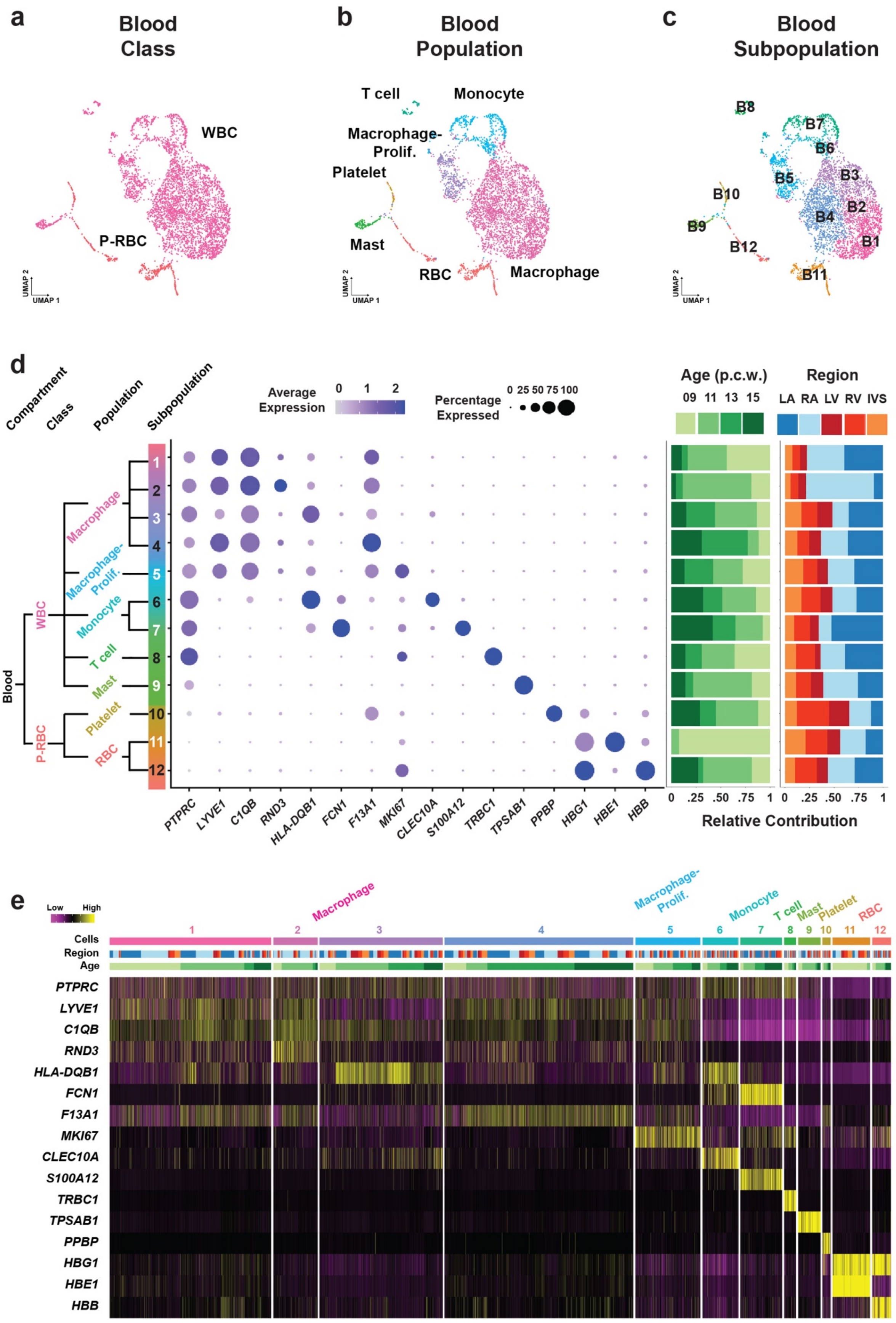
a
Blood Class
WBC
P-RBC
b
Blood Population
T cell
Monocyte
Macrophage-Prolif.
Platelet
Mast
RBC
Macrophage
c
Blood Subpopulation
B1
B2
B3
B4
B5
B6
B7
B8
B9
B10
B11
B12
UMAP 1
UMAP 2
d
Compartment
Class
Population
Subpopulation
Average Expression
Percentage Expressed
0 25 50 75 100
Age (p.c.w.)
09 11 13 15
Region
LA RA LV RV IVS
Blood
WBC
P-RBC
Macrophage
Macrophage-Prolif.
Monocyte
T cell
Mast
Platelet
RBC
PTPRC
LYVE1
C1QB
RND3
HLA-DQB1
FCN1
F13A1
MKI67
CLEC10A
S100A12
TRBC1
TPSAB1
PPBP
HBG1
HBE1
HBB
0 .25 .50 .75 1
Relative Contribution
e
Low
High
Cells
Region
Age
Macrophage
Macrophage-Prolif.
Monocyte
T cell
Mast
Platelet
RBC

Supplementary Figure 7. The blood compartment was present throughout all regions of the human heart, with macrophages representing the largest proportion of blood lineages. **a**, The blood compartment comprises two classes as displayed in UMAP. Each blood class was further subdivided into specific blood cell **b**, populations and **c**, subpopulations. **d**, Dot plot of key marker genes identifies each distinct blood cell and their distribution across age and region - 2 classes, 7 populations, and 12 subpopulations. **e**, Heatmap shows differentially expressed genes identified for each specific blood cell ordered by cell identity, region and age along the x-axis. IVS, interventricular septum; LA, left atrium; LV, left ventricle; p.c.w., post conception weeks; P-RBC, platelet-red blood cell; Prolif., proliferating; RA, right atrium; RBC, red blood cell; RV, right ventricle; WBC, white blood cell.

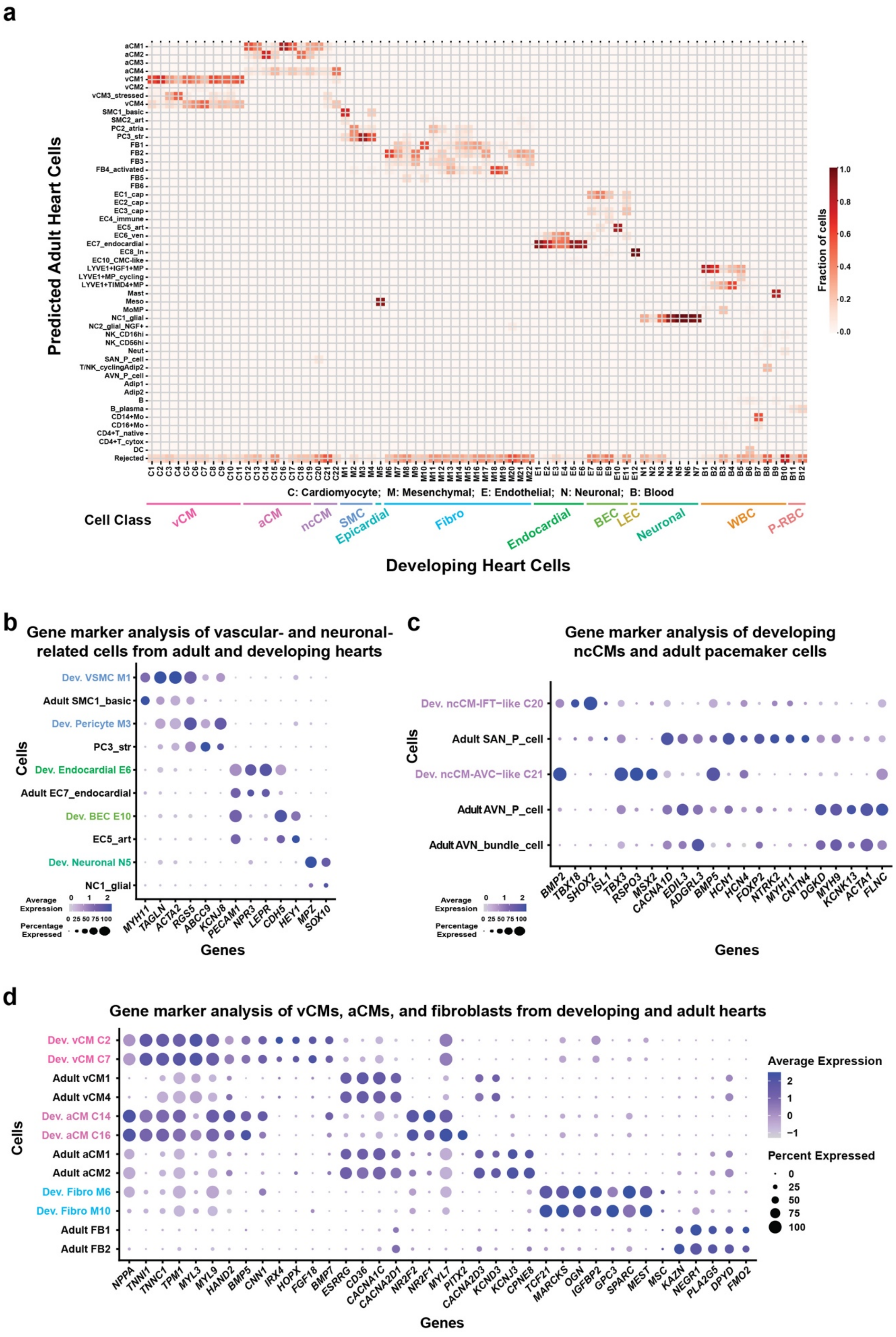
a
Predicted Adult Heart Cells
aCM1
aCM2
aCM3
aCM4
vCM1
vCM2
vCM3_stressed
vCM4
SMC1_basic
SMC2_art
PC2_atria
PC3_str
FB1
FB2
FB3
FB4_activated
FB5
FB6
EC1_cap
EC2_cap
EC3_cap
EC4_immune
EC5_art
EC6_ven
EC7_endocardial
EC8_ln
EC10_CMC-like
LYVE1+IGF1+MP
LYVE1+MP_cycling
LYVE1+TIMD4+MP
Mast
Meso
MoMP
NC1_glial
NC2_glial_NGF+
NK_CD16hi
NK_CD56hi
Neut
SAN_P_cell
T/NK_cycling
Adip2
AVN_P_cell
Adip1
Adip2
B
B_plasma
CD14+Mo
CD16+Mo
CD4+T_native
CD4+T_cytox
DC
Rejected
Fraction of cells
1.0
0.8
0.6
0.4
0.2
0.0
C: Cardiomyocyte; M: Mesenchymal; E: Endothelial; N: Neuronal; B: Blood
Cell Class
vCM
aCM
ncCM
SMC
Epicardial
Fibro
Endocardial
BEC
LEC
Neuronal
WBC
P-RBC
Developing Heart Cells
b
Gene marker analysis of vascular- and neuronal-related cells from adult and developing hearts
Cells
Dev. VSMC M1
Adult SMC1_basic
Dev. Pericyte M3
PC3_str
Dev. Endocardial E6
Adult EC7_endocardial
Dev. BEC E10
EC5_art
Dev. Neuronal N5
NC1_glial
Average Expression
0 1 2
Percentage Expressed
0 25 50 75 100
MYH11
TAGLN
ACTA2
RGS5
ABCC9
KCNJ8
PECAM1
NPR3
LEPR
CDH5
HEY1
MPZ
SOX10
Genes
c
Gene marker analysis of developing ncCMs and adult pacemaker cells
Cells
Dev. ncCM-IFT−like C20
Adult SAN_P_cell
Dev. ncCM-AVC−like C21
Adult AVN_P_cell
Adult AVN_bundle_cell
Average Expression
0 1 2
Percentage Expressed
0 25 50 75 100
BMP2
TBX18
SHOX2
ISL1
TBX3
RSPO3
MSX2
CACNA1D
EDIL3
ADGRL3
BMP5
HCN1
HCN4
FOXP2
NTRK2
MYH11
CNTN4
DGKD
MYH9
KCNK13
ACTA1
FLNC
Genes
d
Gene marker analysis of vCMs, aCMs, and fibroblasts from developing and adult hearts
Cells
Dev. vCM C2
Dev. vCM C7
Adult vCM1
Adult vCM4
Dev. aCM C14
Dev. aCM C16
Adult aCM1
Adult aCM2
Dev. Fibro M6
Dev. Fibro M10
Adult FB1
Adult FB2
Average Expression
2
1
0
−1
Percent Expressed
0
25
50
75
100
NPPA
TNNI1
TNNC1
TPM1
MYL3
MYL9
HAND2
BMP5
CNN1
IRX4
HOPX
FGF18
BMP7
ESRRG
CD36
CACNA1C
CACNA2D1
NR2F2
NR2F1
MYL7
PITX2
CACNA2D3
KCND3
KCNJ3
CPNE8
TCF21
MARCKS
OGN
IGFBP2
GPC3
SPARC
MEST
MSC
KAZN
NEGR1
PLA2G5
DPYD
FMO2
Genes

Supplementary Figure 8. Comparative analysis of our identified developing human cardiac cells with those identified and annotated from adult human hearts reveals cells that are specific to the developing heart. a, Label transfer analyses of cardiac cells between our developing heart cell atlas and Kanemaru et al. adult heart cell atlas show the fraction of developing cells that are assigned to each adult heart cell label utilizing scArches. **b**, Expression dot plot shows examples of vascular- and neuronal-related cells from developing and adult hearts which display similar expression levels of marker genes. **c**, Expression dot plot comparing developing *BMP2*+ non-chamber cardiomyocytes (ncCM) and adult pacemaker cells shows that developing *BMP2*+ ncCMs express higher levels of transcription factors than adult pacemaker cells (e.g., *SHOX2, TBX18, MSX2*), while adult pacemaker cells express higher levels of ion channels than developing *BMP2*+ ncCMs (e.g., *HCN1, CACNA1D*). **d**, Expression dot plot shows examples of gene expression differences between corresponding adult and developing vCMs, aCMs, and cardiac fibroblasts. Developing vCMs and aCMs express higher levels of known fetal cardiac genes than adult vCMs and aCMs, respectively, as previously reported (*NPPA*[29], *TNNI1*[30], *IRX4*[31]), while developing cardiac fibroblasts express the known developmental regulator *TCF21*[32] and higher levels of specific extracellular matrix-related proteins than adult cardiac fibroblasts (*SPARC, GPC3*). aCM, atrial cardiomyocyte; AVC, atrioventricular canal; AVN, atrioventricular node; BEC, blood endothelial cell; Dev., developing; EC, endothelial cell; FB, fibroblast; Fibro, fibroblast; IFT, inflow tract; NC, neuronal cell; ncCM, non-chambered cardiomyocyte; PC, pericyte; P cell, pacemaker cell; SAN, sinoatrial node; SMC, smooth muscle cell; vCM, ventricular cardiomyocyte; VSMC, vascular smooth muscle cell.

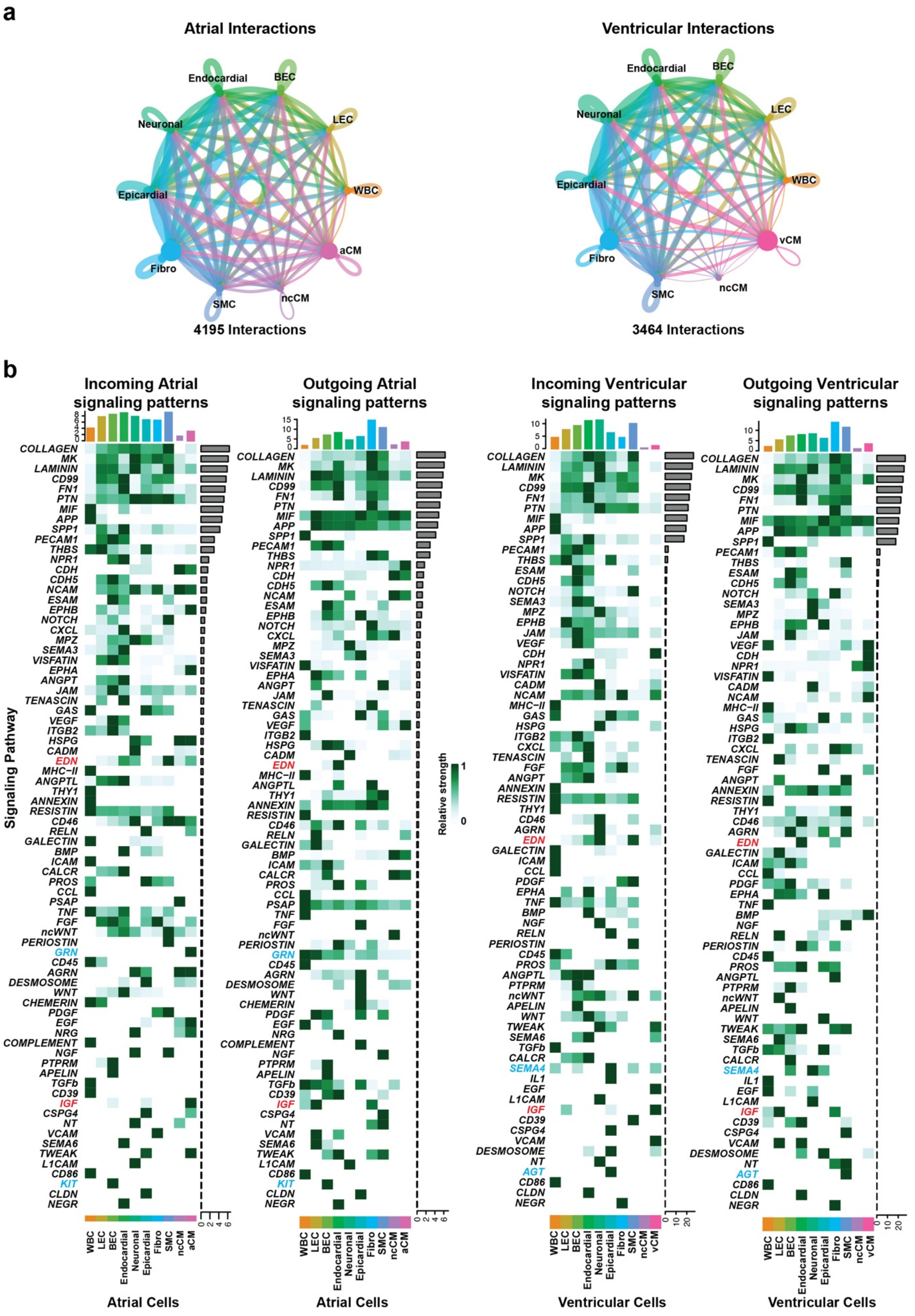
a
Atrial Interactions
Endocardial
BEC
LEC
Neuronal
WBC
Epicardial
aCM
Fibro
SMC
ncCM
4195 Interactions
Ventricular Interactions
Endocardial
BEC
LEC
Neuronal
WBC
Epicardial
vCM
Fibro
SMC
ncCM
3464 Interactions
b
Incoming Atrial signaling patterns
Outgoing Atrial signaling patterns
Incoming Ventricular signaling patterns
Outgoing Ventricular signaling patterns
Signaling Pathway
COLLAGEN MK LAMININ CD99 FN1 PTN MIF APP SPP1 PECAM1 THBS NPR1 CDH CDH5 NCAM ESAM EPHB NOTCH CXCL MPZ SEMA3 VISFATIN EPHA ANGPT JAM TENASCIN GAS VEGF ITGB2 HSPG CADM EDN MHC−II ANGPTL THY1 ANNEXIN RESISTIN CD46 RELN GALECTIN BMP ICAM CALCR PROS CCL PSAP TNF FGF ncWNT PERIOSTIN GRN CD45 AGRN DESMOSOME WNT CHEMERIN PDGF EGF NRG COMPLEMENT NGF PTPRM APELIN TGFb CD39 IGF CSPG4 NT VCAM SEMA6 TWEAK L1CAM CD86 KIT CLDN NEGR
COLLAGEN MK LAMININ CD99 FN1 PTN MIF APP SPP1 PECAM1 THBS NPR1 CDH CDH5 NCAM ESAM EPHB NOTCH CXCL MPZ SEMA3 VISFATIN EPHA ANGPT JAM TENASCIN GAS VEGF ITGB2 HSPG CADM EDN MHC−II ANGPTL THY1 ANNEXIN RESISTIN CD46 RELN GALECTIN BMP ICAM CALCR PROS CCL PSAP TNF FGF ncWNT PERIOSTIN GRN CD45 AGRN DESMOSOME WNT CHEMERIN PDGF EGF NRG COMPLEMENT NGF PTPRM APELIN TGFb CD39 IGF CSPG4 NT VCAM SEMA6 TWEAK L1CAM CD86 KIT CLDN NEGR
COLLAGEN LAMININ MK CD99 FN1 PTN MIF APP SPP1 PECAM1 THBS ESAM CDH5 NOTCH SEMA3 MPZ EPHB JAM VEGF CDH NPR1 VISFATIN CADM NCAM MHC−II GAS HSPG ITGB2 CXCL TENASCIN FGF ANGPT ANNEXIN RESISTIN THY1 CD46 AGRN EDN GALECTIN ICAM CCL PDGF EPHA TNF BMP NGF RELN PERIOSTIN CD45 PROS ANGPTL PTPRM ncWNT APELIN WNT TWEAK SEMA6 TGFb CALCR SEMA4 IL1 EGF L1CAM IGF CD39 CSPG4 VCAM DESMOSOME NT AGT CD86 CLDN NEGR
COLLAGEN LAMININ MK CD99 FN1 PTN MIF APP SPP1 PECAM1 THBS ESAM CDH5 NOTCH SEMA3 MPZ EPHB JAM VEGF CDH NPR1 VISFATIN CADM NCAM MHC−II GAS HSPG ITGB2 CXCL TENASCIN FGF ANGPT ANNEXIN RESISTIN THY1 CD46 AGRN EDN GALECTIN ICAM CCL PDGF EPHA TNF BMP NGF RELN PERIOSTIN CD45 PROS ANGPTL PTPRM ncWNT APELIN WNT TWEAK SEMA6 TGFb CALCR SEMA4 IL1 EGF L1CAM IGF CD39 CSPG4 VCAM DESMOSOME NT AGT CD86 CLDN NEGR
Relative strength
1
0
WBC LEC BEC Endocardial Neuronal Epicardial Fibro SMC ncCM aCM
Atrial Cells
WBC LEC BEC Endocardial Neuronal Epicardial Fibro SMC ncCM aCM
Atrial Cells
WBC LEC BEC Endocardial Neuronal Epicardial Fibro SMC ncCM vCM
Ventricular Cells
WBC LEC BEC Endocardial Neuronal Epicardial Fibro SMC ncCM vCM
Ventricular Cells

Supplementary Figure 9. Cell-cell signaling analysis reveals distinct cell signaling interactions within early developing human atria and ventricles. a, Chord diagrams reveal distinct multi-cellular signaling interactions within the atria (left) and ventricles (right) of developing human hearts. The size of the node represents the number of cells for a distinct atrial/ventricular cell, and the width of the edge represents the number of interactions between pairs of distinct atrial/ventricular cells. **b,** Heatmaps show the signaling strength of each signaling pathway for different pairs of distinct cells within the atria (left two heatmaps) and ventricles (right two heatmaps). Signaling pathways are ordered by overall strength of signaling. For each heatmap pair, the left heatmap represents outgoing (ligand) signaling, and the right one represents incoming (receptor) signaling. Examples of shared signaling pathways are labeled in red, while examples of distinct signaling pathways are labeled in light blue. aCM, atrial cardiomyocyte; BEC, blood endothelial cell; Fibro, fibroblast; LEC, lymphatic endothelial cell; ncCM, non-chambered cardiomyocyte; SMC, smooth muscle cell; vCM, ventricular cardiomyocyte; WBC, white blood cell.

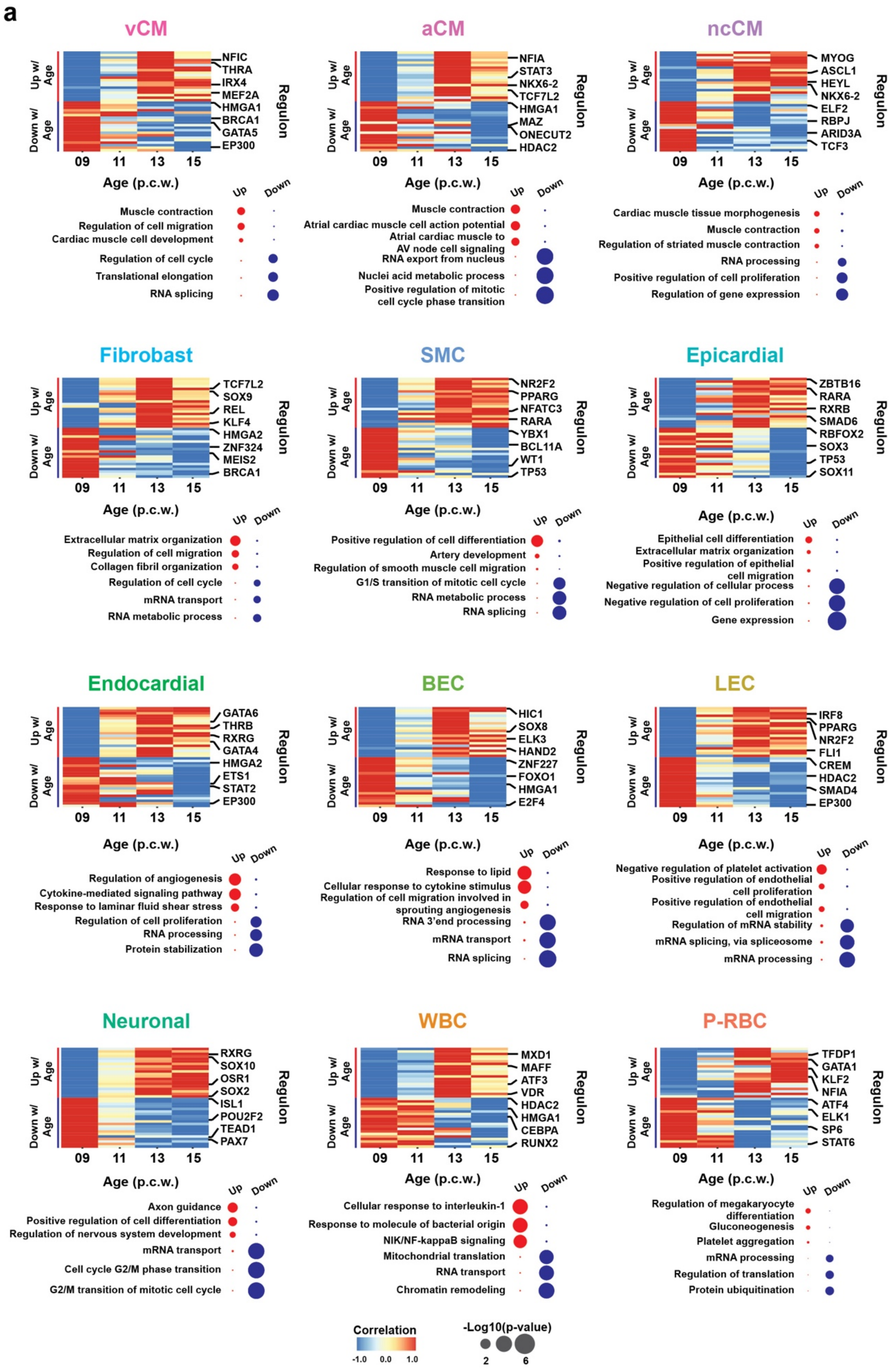

a
vCM
NFIC
THRA
IRX4
MEF2A
HMGA1
BRCA1
GATA5
EP300
Up w/ Age
Down w/ Age
Regulon
09 11 13 15
Age (p.c.w.)
Up Down
Muscle contraction
Regulation of cell migration
Cardiac muscle cell development
Regulation of cell cycle
Translational elongation
RNA splicing
aCM
NFIA
STAT3
NKX6-2
TCF7L2
HMGA1
MAZ
ONECUT2
HDAC2
Muscle contraction
Atrial cardiac muscle cell action potential
Atrial cardiac muscle to AV node cell signaling
RNA export from nucleus
Nuclei acid metabolic process
Positive regulation of mitotic cell cycle phase transition
ncCM
MYOG
ASCL1
HEYL
NKX6-2
ELF2
RBPJ
ARID3A
TCF3
Cardiac muscle tissue morphogenesis
Muscle contraction
Regulation of striated muscle contraction
RNA processing
Positive regulation of cell proliferation
Regulation of gene expression
Fibrobast
TCF7L2
SOX9
REL
KLF4
HMGA2
ZNF324
MEIS2
BRCA1
Extracellular matrix organization
Regulation of cell migration
Collagen fibril organization
Regulation of cell cycle
mRNA transport
RNA metabolic process
SMC
NR2F2
PPARG
NFATC3
RARA
YBX1
BCL11A
WT1
TP53
Positive regulation of cell differentiation
Artery development
Regulation of smooth muscle cell migration
G1/S transition of mitotic cell cycle
RNA metabolic process
RNA splicing
Epicardial
ZBTB16
RARA
RXRB
SMAD6
RBFOX2
SOX3
TP53
SOX11
Epithelial cell differentiation
Extracellular matrix organization
Positive regulation of epithelial cell migration
Negative regulation of cellular process
Negative regulation of cell proliferation
Gene expression
Endocardial
GATA6
THRB
RXRG
GATA4
HMGA2
ETS1
STAT2
EP300
Regulation of angiogenesis
Cytokine-mediated signaling pathway
Response to laminar fluid shear stress
Regulation of cell proliferation
RNA processing
Protein stabilization
BEC
HIC1
SOX8
ELK3
HAND2
ZNF227
FOXO1
HMGA1
E2F4
Response to lipid
Cellular response to cytokine stimulus
Regulation of cell migration involved in sprouting angiogenesis
RNA 3'end processing
mRNA transport
RNA splicing
LEC
IRF8
PPARG
NR2F2
FLI1
CREM
HDAC2
SMAD4
EP300
Negative regulation of platelet activation
Positive regulation of endothelial cell proliferation
Positive regulation of endothelial cell migration
Regulation of mRNA stability
mRNA splicing, via spliceosome
mRNA processing
Neuronal
RXRG
SOX10
OSR1
SOX2
ISL1
POU2F2
TEAD1
PAX7
Axon guidance
Positive regulation of cell differentiation
Regulation of nervous system development
mRNA transport
Cell cycle G2/M phase transition
G2/M transition of mitotic cell cycle
WBC
MXD1
MAFF
ATF3
VDR
HDAC2
HMGA1
CEBPA
RUNX2
Cellular response to interleukin-1
Response to molecule of bacterial origin
NIK/NF-kappaB signaling
Mitochondrial translation
RNA transport
Chromatin remodeling
P-RBC
TFDP1
GATA1
KLF2
NFIA
ATF4
ELK1
SP6
STAT6
Regulation of megakaryocyte differentiation
Gluconeogenesis
Platelet aggregation
mRNA processing
Regulation of translation
Protein ubiquitination
Correlation
-1.0 0.0 1.0
-Log10(p-value)
2 6

Supplementary Figure 10. Gene regulatory network analysis utilizing pySCENIC reveals age-related developmental regulons for each distinct cell. a, The top 20 regulons that positively and negatively correlate with age are shown in heatmap (top). The GO enrichment analysis of the genes for these modules are provided below each heatmap (bottom). aCM, atrial cardiomyocyte; BEC, blood endothelial cell; LEC, lymphatic endothelial cell; ncCM, non-chambered cardiomyocyte; p.c.w., post conception weeks; P-RBC, platelet-red blood cell; SMC, smooth muscle cell; vCM, ventricular cardiomyocyte; WBC, white blood cell.

a

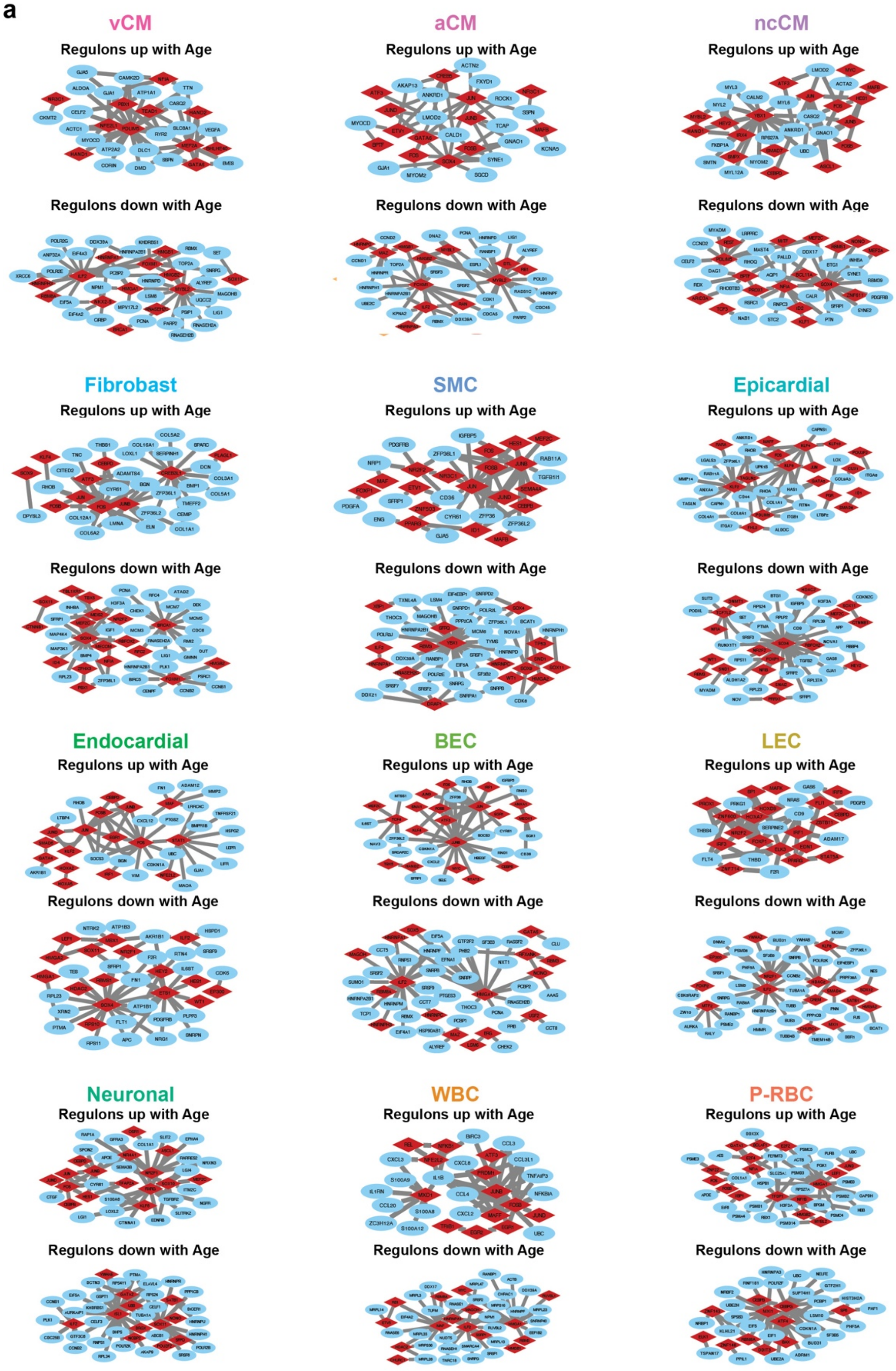
vCM
Regulons up with Age
Regulons down with Age
aCM
Regulons up with Age
Regulons down with Age
ncCM
Regulons up with Age
Regulons down with Age
Fibrobast
Regulons up with Age
Regulons down with Age
SMC
Regulons up with Age
Regulons down with Age
Epicardial
Regulons up with Age
Regulons down with Age
Endocardial
Regulons up with Age
Regulons down with Age
BEC
Regulons up with Age
Regulons down with Age
LEC
Regulons up with Age
Regulons down with Age
Neuronal
Regulons up with Age
Regulons down with Age
WBC
Regulons up with Age
Regulons down with Age
P-RBC
Regulons up with Age
Regulons down with Age

Supplementary Figure 11. Gene network visualization of pySCENIC regulons reveals potential regulators of age-related developmental gene modules for each distinct cell. a, Network plots of genes and transcription factors (TFs) assigned to the age-correlated regulons from the pySCENIC analysis reveal the co-regulated relationship between individual genes/TFs in distinct cells, and potential transcriptional regulators of age-related regulons. Red diamonds represent TFs and blue ovals represent non-TFs. aCM, atrial cardiomyocyte; BEC, blood endothelial cell; LEC, lymphatic endothelial cell; ncCM, non-chambered cardiomyocyte; P-RBC, platelet-red blood cell; SMC, smooth muscle cell; vCM, ventricular cardiomyocyte; WBC, white blood cell.

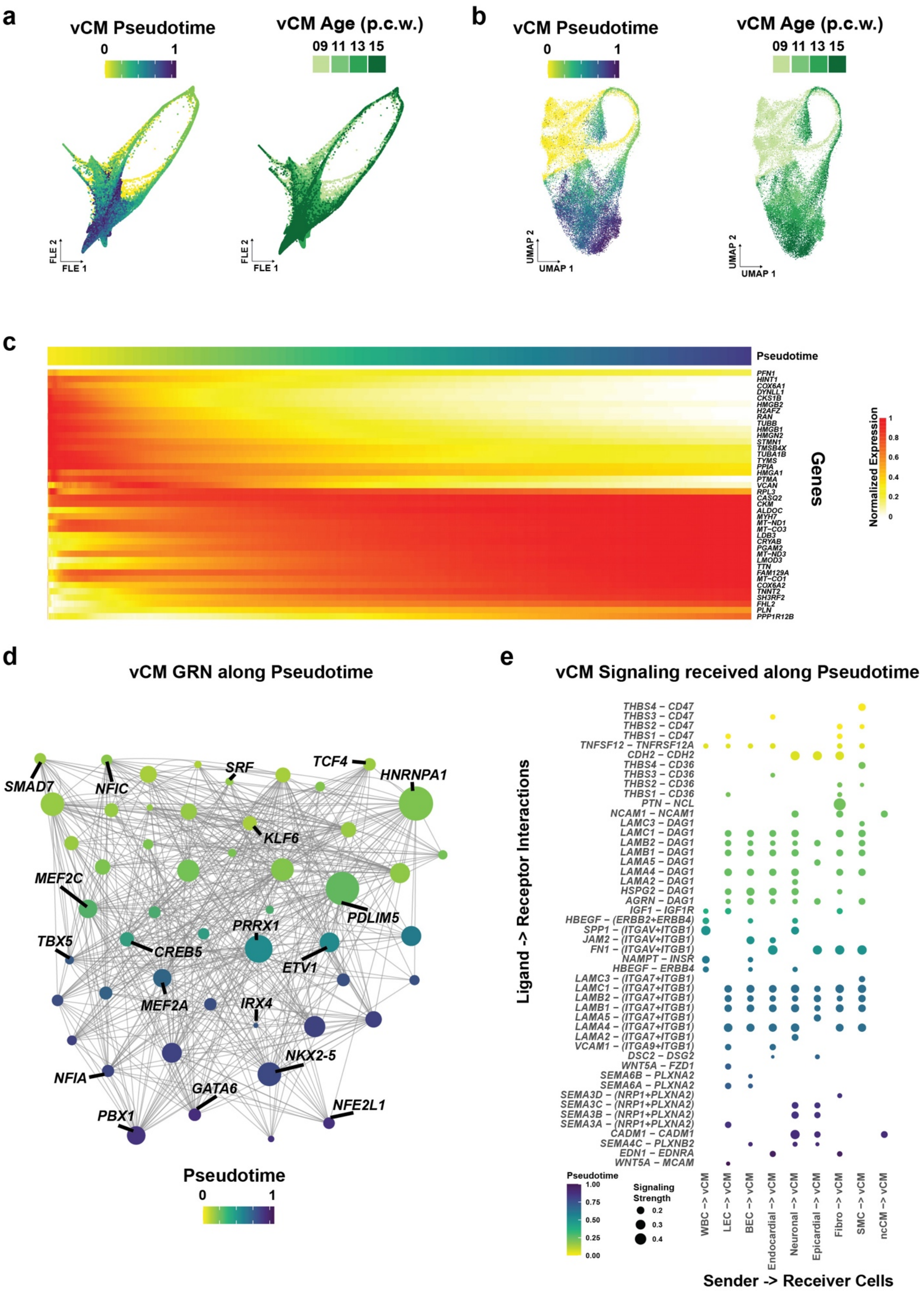
a
vCM Pseudotime
0
1
vCM Age (p.c.w.)
09 11 13 15
FLE 2
FLE 1
b
vCM Pseudotime
0
1
vCM Age (p.c.w.)
09 11 13 15
UMAP 2
UMAP 1
c
Pseudotime
PFN1
HINT1
COX6A1
DYNLL1
CKS1B
HMGB2
H2AFZ
RAN
TUBB
HMGB1
HMGN2
STMN1
TMSB4X
TUBA1B
TYMS
PPIA
HMGA1
PTMA
VCAN
RPL3
CASQ2
CKM
ALDOC
MYH7
MT−ND1
MT−CO3
LDB3
CRYAB
PGAM2
MT−ND3
LMOD3
TTN
FAM129A
MT−CO1
COX6A2
TNNT2
SH3RF2
FHL2
PLN
PPP1R12B
Genes
Normalized Expression
1
0.8
0.6
0.4
0.2
0
d
vCM GRN along Pseudotime
SMAD7
NFIC
SRF
TCF4
HNRNPA1
KLF6
MEF2C
PDLIM5
TBX5
CREB5
PRRX1
ETV1
MEF2A
IRX4
NKX2-5
NFIA
GATA6
NFE2L1
PBX1
Pseudotime
0
1
e
vCM Signaling received along Pseudotime
THBS4 − CD47
THBS3 − CD47
THBS2 − CD47
THBS1 − CD47
TNFSF12 − TNFRSF12A
CDH2 − CDH2
THBS4 − CD36
THBS3 − CD36
THBS2 − CD36
THBS1 − CD36
PTN − NCL
NCAM1 − NCAM1
LAMC3 − DAG1
LAMC1 − DAG1
LAMB2 − DAG1
LAMB1 − DAG1
LAMA5 − DAG1
LAMA4 − DAG1
LAMA2 − DAG1
HSPG2 − DAG1
AGRN − DAG1
IGF1 − IGF1R
HBEGF − (ERBB2+ERBB4)
SPP1 − (ITGAV+ITGB1)
JAM2 − (ITGAV+ITGB1)
FN1 − (ITGAV+ITGB1)
NAMPT − INSR
HBEGF − ERBB4
LAMC3 − (ITGA7+ITGB1)
LAMC1 − (ITGA7+ITGB1)
LAMB2 − (ITGA7+ITGB1)
LAMB1 − (ITGA7+ITGB1)
LAMA5 − (ITGA7+ITGB1)
LAMA4 − (ITGA7+ITGB1)
LAMA2 − (ITGA7+ITGB1)
VCAM1 − (ITGA9+ITGB1)
DSC2 − DSG2
WNT5A − FZD1
SEMA6B − PLXNA2
SEMA6A − PLXNA2
SEMA3D − (NRP1+PLXNA2)
SEMA3C − (NRP1+PLXNA2)
SEMA3B − (NRP1+PLXNA2)
SEMA3A − (NRP1+PLXNA2)
CADM1 − CADM1
SEMA4C − PLXNB2
EDN1 − EDNRA
WNT5A − MCAM
Ligand -> Receptor Interactions
Pseudotime
1.00
0.75
0.50
0.25
0.00
Signaling Strength
0.2
0.3
0.4
WBC −> vCM
LEC −> vCM
BEC −> vCM
Endocardial −> vCM
Neuronal −> vCM
Epicardial −> vCM
Fibro −> vCM
SMC −> vCM
ncCM −> vCM
Sender -> Receiver Cells

Supplementary Figure 12. vCM trajectory analysis reveals the gene regulatory and cell signaling dynamics that may influence vCM development. a, A trajectory analysis as displayed by a force directed graph was performed on vCMs utilizing Waddington-OT and colored by pseudotime (left plot) and age (right plot). **b**, Waddington-OT pseudotime was projected onto the UMAP embedding of vCMs (left plot), and shows a correlation with developmental age progression (right plot). **c,** Heatmap of vCMs ordered by pseudotime shows the top genes that increase and decrease with pseudotime. **d,** The transcription factors (TFs) within the pySCENIC-derived vCM gene regulatory network were ordered by pseudotime from the trajectory analysis in order to reveal the gene regulatory dynamics governing vCM development. The size of the node represents TF centrality in the network. **e,** Dot plot shows the interactions received by vCMs from other cells. These interactions were ordered by pseudotime from the trajectory analysis to illuminate potential interactions that may be important for early vs late stages of vCM development. The size of the dots indicates the signaling strength of interactions which is based on the expression of the ligand and cognate receptor. BEC, blood endothelial cell; Fibro, fibroblast; LEC, lymphatic endothelial cell; ncCM, non-chambered cardiomyocyte; p.c.w., post conception weeks; SMC, smooth muscle cell; vCM, ventricular cardiomyocyte; WBC, white blood cell.

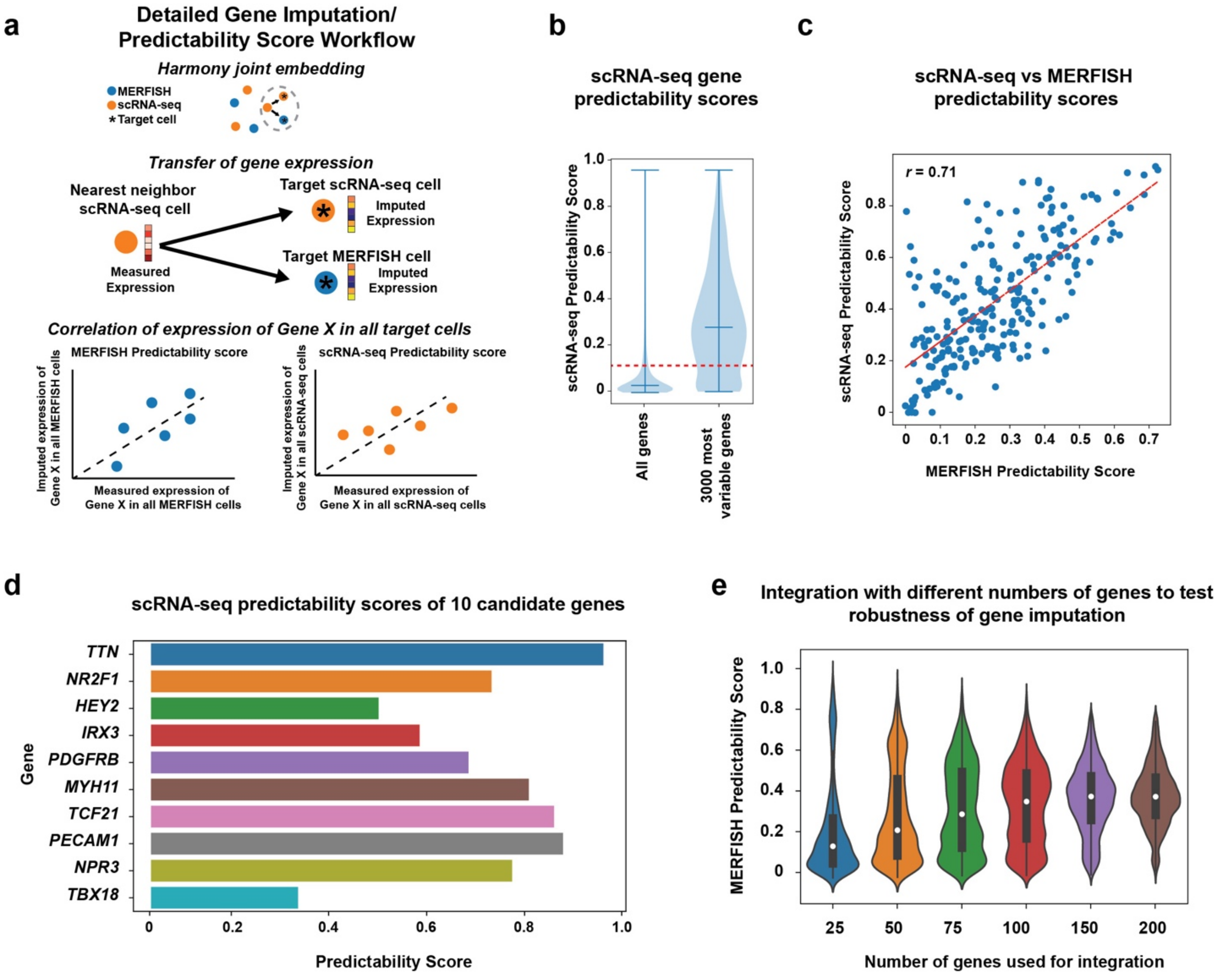

Supplementary Figure 13. Joint embedding analysis of scRNA-seq and MERFISH datasets enables imputation of the expression of cardiac genes. a, Diagram illustrates how gene imputation is determined and scored for predictability based on a Pearson correlation between the imputed and measured expression of a specific gene for an individual cell. **b**, scRNA-seq predictability scores of gene expression for each scRNA-seq cell with its nearest neighbor scRNA-seq cell in the Harmony integrated PCA space reveals lower predictability scores using all 26,581 genes (median = 0.024) compared to those using the 3,000 most variable genes (median = 0.276). The dashed

red line indicates the threshold (scRNA-seq predictability score = 0.11) above which all predictability scores show statistically significant improvement over random prediction (Bonferroni adjusted p-value < 0.01). The center blue line represents the median, the other two blue lines define minima and maxima of the distribution. **c**, Scatterplot reveals correlation between scRNA-seq and MERFISH predictability scores (Pearson correlation $r = 0.71$), which indicates that genes that can be predicted with high confidence with scRNA-seq can also be predicted with high confidence in the MERFISH dataset. **d**, The gene predictability scores calculated for ten marker genes corresponding to the spatial expression plots in Fig. 1g supports that the gene imputation can confidently map gene expression. **e**, Violin plots show that increasing the number of genes used for the joint embedding integration improves predictability scores up to 150 genes, after which the predictability scores plateau. The center white dot represents the median, the bold black line represents the interquartile range, and the edges define minima and maxima of the distribution. N = 238 genes per group.

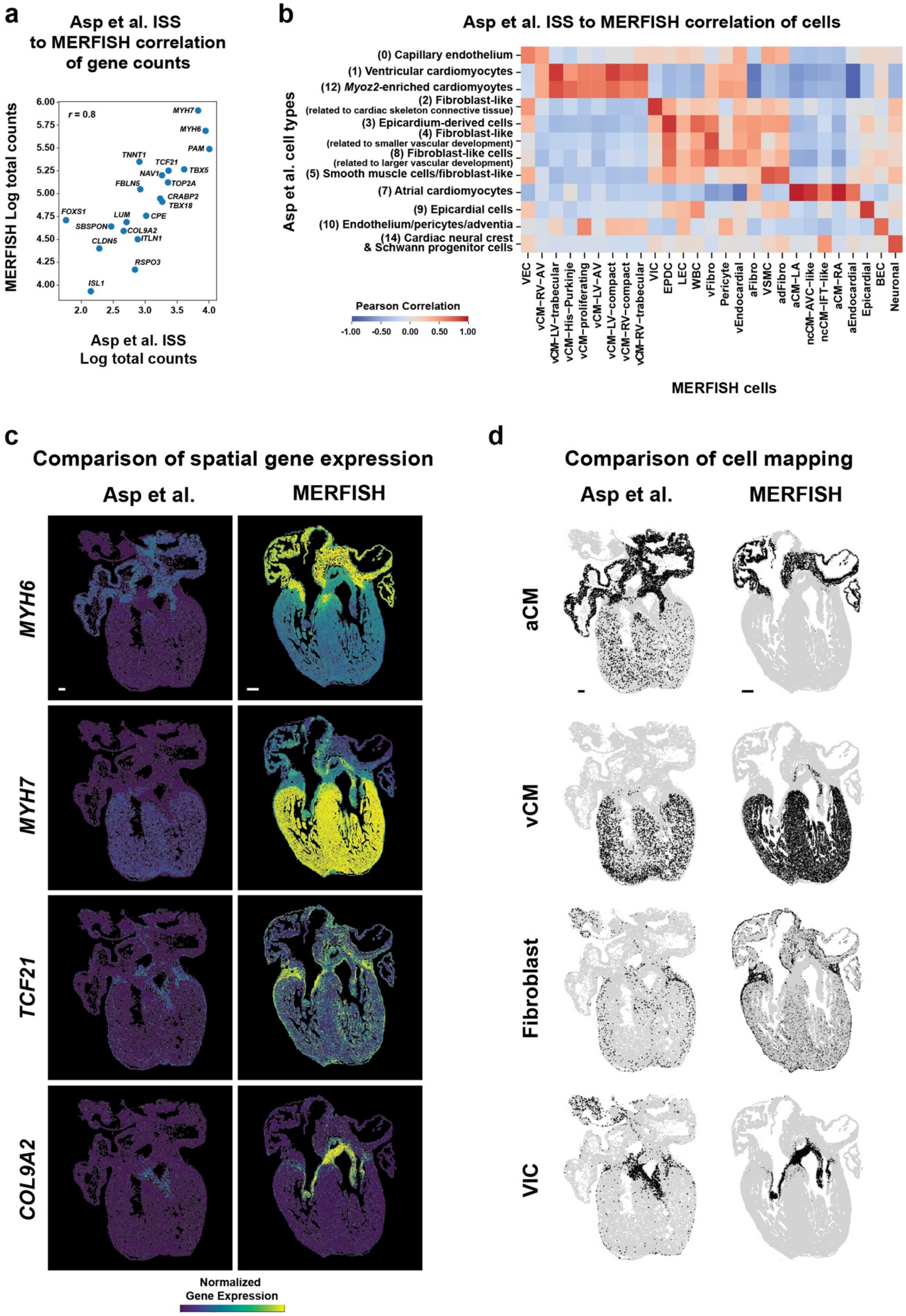
a
Asp et al. ISS to MERFISH correlation of gene counts
r = 0.8
MERFISH Log total counts
Asp et al. ISS Log total counts
MYH7
MYH6
PAM
TNNT1
TCF21
TBX5
NAV1
TOP2A
FBLN5
CRABP2
TBX18
FOXS1
LUM
CPE
SBSPON
COL9A2
CLDN5
ITLN1
RSPO3
ISL1
b
Asp et al. ISS to MERFISH correlation of cells
Asp et al. cell types
(0) Capillary endothelium
(1) Ventricular cardiomyocytes
(12) Myoz2-enriched cardiomyoytes
(2) Fibroblast-like (related to cardiac skeleton connective tissue)
(3) Epicardium-derived cells
(4) Fibroblast-like (related to smaller vascular development)
(8) Fibroblast-like cells (related to larger vascular development)
(5) Smooth muscle cells/fibroblast-like
(7) Atrial cardiomyocytes
(9) Epicardial cells
(10) Endothelium/pericytes/adventia
(14) Cardiac neural crest & Schwann progenitor cells
Pearson Correlation
-1.00 -0.50 0.00 0.50 1.00
VEC
vCM−RV−AV
vCM−LV−trabecular
vCM−His−Purkinje
vCM−proliferating
vCM−LV−AV
vCM−LV−compact
vCM−RV−compact
vCM−RV−trabecular
VIC
EPDC
LEC
WBC
vFibro
Pericyte
vEndocardial
aFibro
VSMC
adFibro
aCM−LA
ncCM−AVC−like
ncCM−IFT−like
aCM−RA
aEndocardial
Epicardial
BEC
Neuronal
MERFISH cells
c
Comparison of spatial gene expression
Asp et al.
MERFISH
MYH6
MYH7
TCF21
COL9A2
Normalized Gene Expression
d
Comparison of cell mapping
Asp et al.
MERFISH
aCM
vCM
Fibroblast
VIC

Supplementary Figure 14. MERFISH analysis provides additional spatial resolution of cell populations when compared to other developing human heart spatial transcriptomic datasets. a, Pearson correlation (*r*) of the transcript counts for each of the 20 shared target genes between the MERFISH and Asp et al. datasets[1] reveals similar relative expression levels of the target genes between datasets. **b**, Heatmap shows that the MERFISH dataset contained all cardiac cells previously identified by Asp et al. dataset, as well as additional new distinct cardiac cell populations. **c**, Spatial expression plots of example key marker genes are shown from both datasets. **d**, The spatial distribution of specific cell populations are shown from both datasets. aCM, atrial cardiomyocyte; aFibro, atrial fibroblast; adFibro, adventitial fibroblast; aEndocardial, atrial endocardial; AVC, atrioventricular canal; BEC, blood endothelial cell; EPDC, epicardial-derived cell; IFT, inflow tract; LA, left atrium; LEC, lymphatic endothelial cell; LV, left ventricle; ncCM, non-chambered cardiomyocyte; RA, right atrium; RV, right ventricle; vCM, ventricular cardiomyocyte; vCM-LV/RV-AV, muscular valve leaflet vCM; vEndocardial, ventricular endocardial; VEC, valve endocardial cell; vFibro, ventricular fibroblast; VIC, valve interstitial cell; VSMC, vascular smooth muscle cell; WBC, white blood cell. Scale bar, 250 µm.

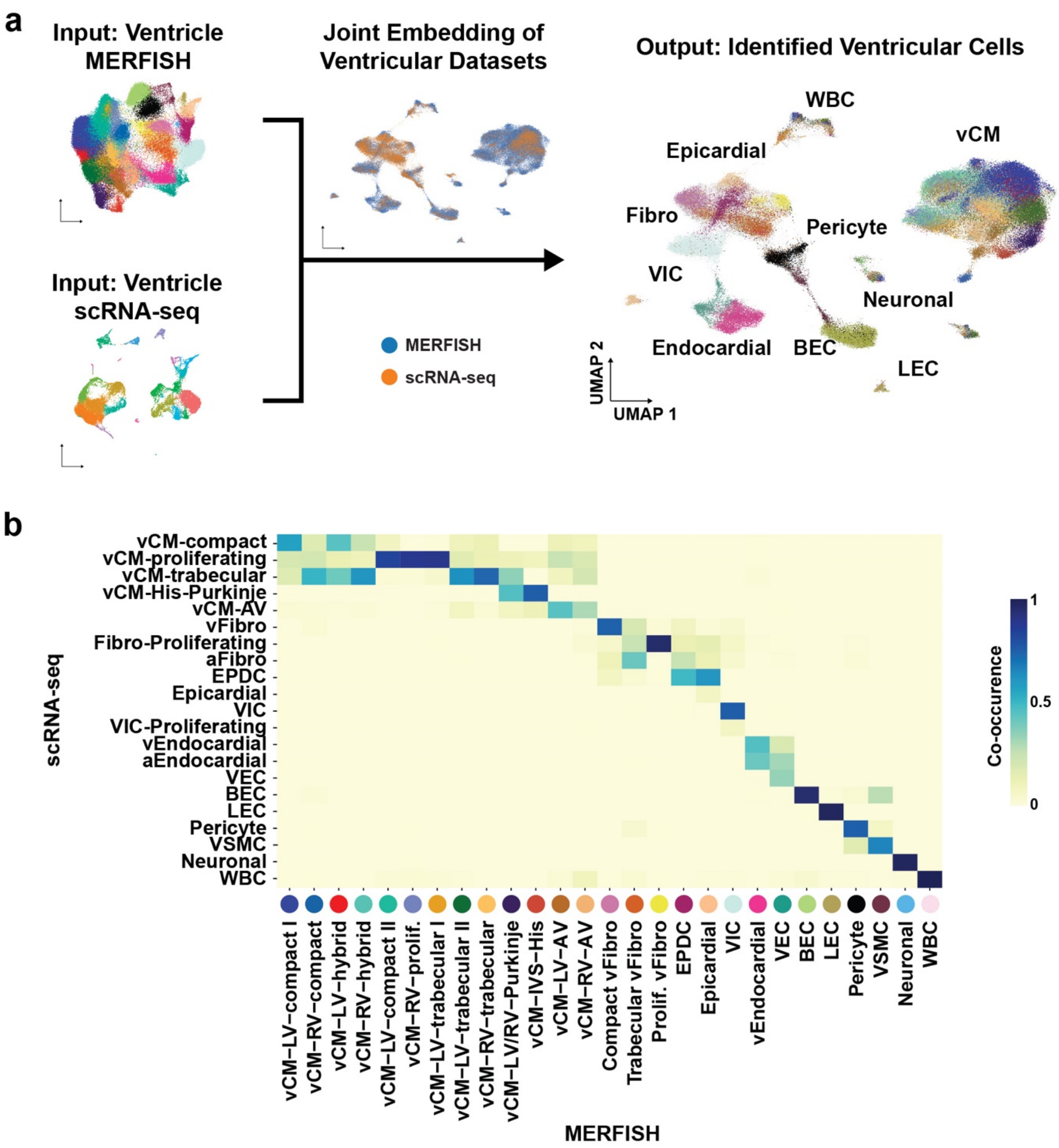

Supplementary Figure 15. Joint embedding of scRNA-seq and MERFISH datasets enables identification of specific MERFISH ventricular cells in the scRNA-seq dataset. a, Joint embedding between scRNA-seq and MERFISH datasets for ventricular cells enabled cell label transfer as shown on UMAP based on identified

ventricular cell. **b**, Co-occurrence heatmap shows the correspondence of MERFISH cell labels between MERFISH ventricular annotations and labels transferred from 13 post conception week scRNA-seq. aFibro, atrial fibroblast; BEC, blood endothelial cell; EPDC, epicardial-derived cell; Fibro, fibroblast; IVS, interventricular septum; LEC, lymphatic endothelial cell; LV, left ventricle; Prolif., proliferating; RV, right ventricle; vCM, ventricular cardiomyocyte; vCM-AV, muscular valve leaflet vCM; vCM-LV/RV-AV, muscular valve leaflet vCM; VEC, valve endocardial cell; vEndocardial, ventricular endocardial; vFibro, ventricular fibroblast; VIC, valve interstitial cell; VSMC, vascular smooth muscle cell; WBC, white blood cell.

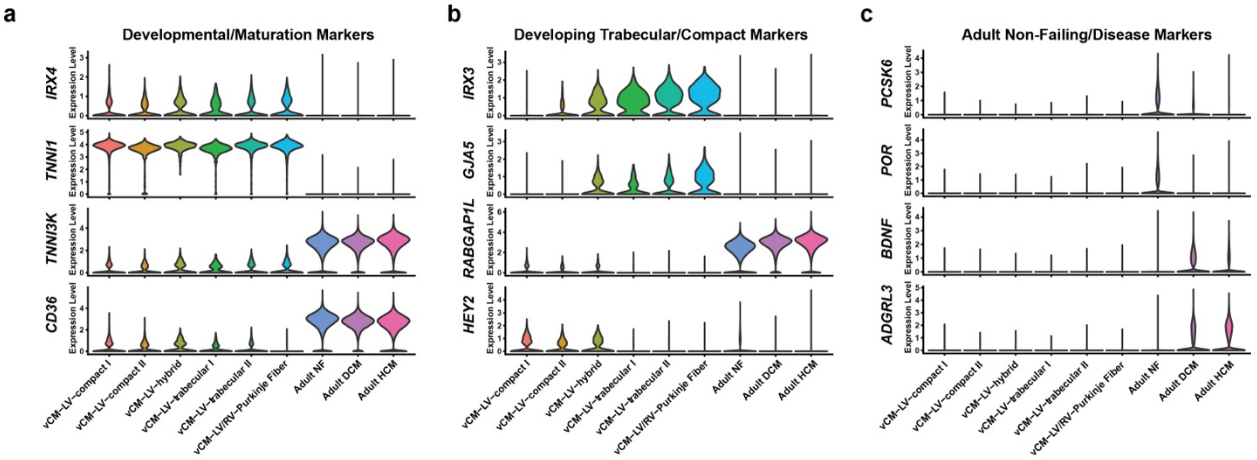

Supplementary Figure 16. The relationship between developing human vCM subpopulations and non-failing/diseased adult human vCMs were examined using cell-type specific gene markers identified from corresponding scRNA-seq data. a, Developing human vCMs but not adult human vCMs express gene markers of early heart development (*IRX4, TNNI1*), whereas adult human vCMs exhibit higher expression of markers of mature sarcomeres (*TNNI3K*) and fatty acid metabolism (*CD36*) than developing human vCMs. **b**, Gene markers specific for trabecular vCMs (*GJA5*, *IRX3*) are present in developing human trabecular and Purkinje fiber vCM subpopulations, but not in adult human vCMs; however, while the compact markers, *RABGAP1L* and *HEY2,* are expressed in developing compact human vCM subpopulations, only *RABGAP1L* is expressed in non-failing/diseased adult human vCMs. **c**, Gene markers distinguishing between non-failing (*PCSK9, POR*) and diseased (*BDNF, ADGRL3*) adult human vCMs are not expressed in developing human vCMs. DCM, dilated cardiomyopathy; HCM, hypertrophic cardiomyopathy; LV, left ventricle; NF, non-failing; vCM, ventricular cardiomyocyte.

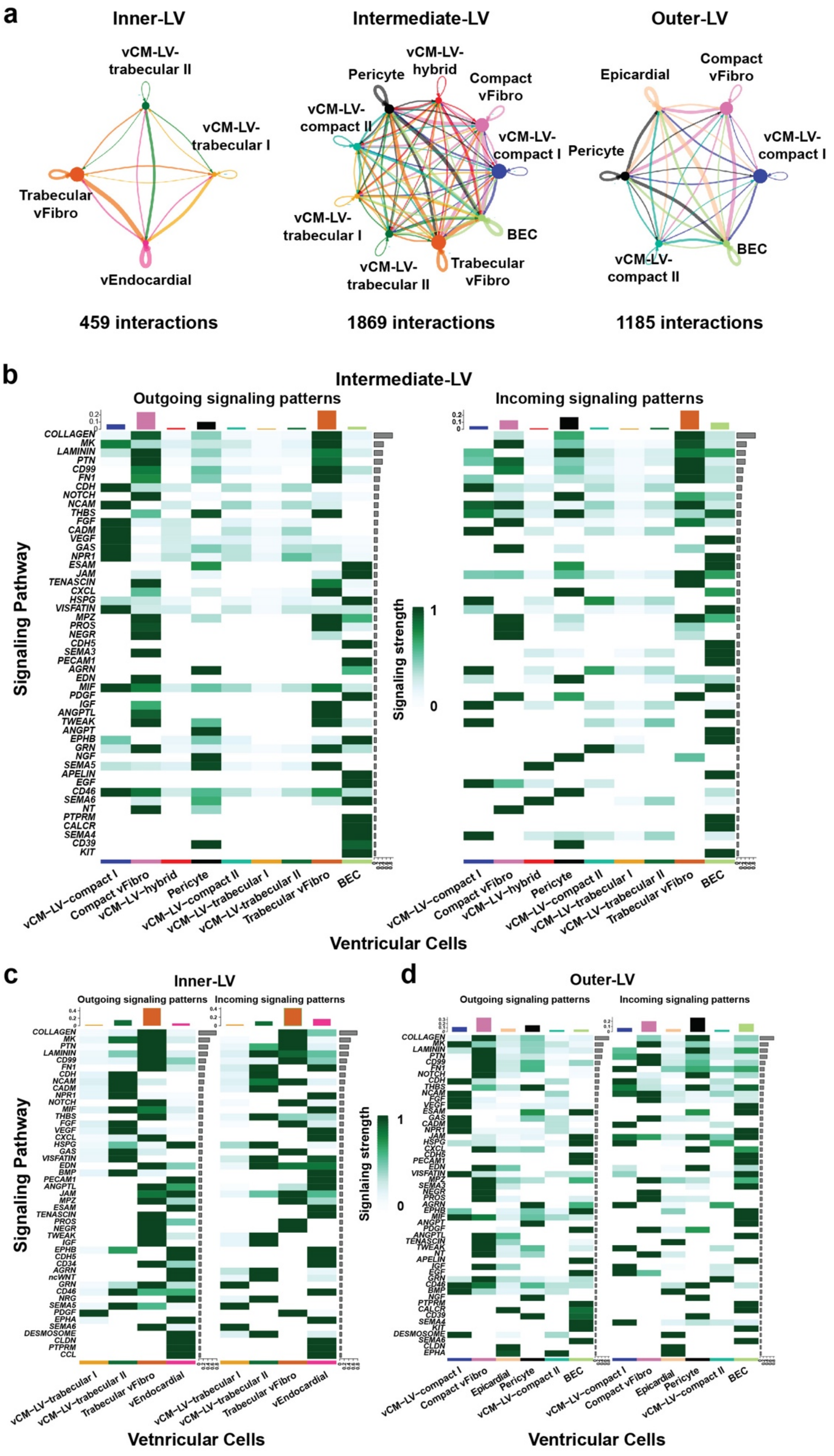
a
Inner-LV
vCM-LV-trabecular II
vCM-LV-trabecular I
Trabecular vFibro
vEndocardial
459 interactions
Intermediate-LV
vCM-LV-hybrid
Pericyte
Compact vFibro
vCM-LV-compact II
vCM-LV-compact I
vCM-LV-trabecular I
BEC
vCM-LV-trabecular II
Trabecular vFibro
1869 interactions
Outer-LV
Epicardial
Compact vFibro
Pericyte
vCM-LV-compact I
BEC
vCM-LV-compact II
1185 interactions
b
Intermediate-LV
Outgoing signaling patterns
Incoming signaling patterns
Signaling Pathway
Signaling strength
Ventricular Cells
c
Inner-LV
Outgoing signaling patterns
Incoming signaling patterns
Signaling Pathway
Vetnricular Cells
d
Outer-LV
Outgoing signaling patterns
Incoming signaling patterns
Signaling strength
Ventricular Cells

Supplementary Figure 17. Cell-cell signaling analysis reveals distinct cell signaling interactions within the Inner-LV, Intermediate-LV, and Outer-LV cell communities. a, Chord diagrams reveal distinct multi-cellular signaling interactions within the Inner-LV, Intermediate-LV, and Outer-LV cell communities of the left ventricle. The size of the node represents the number of cells for a distinct ventricular cell, and the width of the edge represents the number of interactions between pairs of distinct ventricular cells. **b-d,** Heatmaps show the signaling strength of each signaling pathway for different pairs of distinct ventricular cells within the (**b**) Intermediate-LV community, (**c**) Inner-LV community, and (**d**) Outer-LV community. Signaling pathways are ordered by overall strength of signaling. For each heatmap pair, the left heatmap represents outgoing (ligand) signaling, and the right one represents incoming (receptor) signaling. BEC, blood endothelial cell; LV, left ventricle; vCM, ventricular cardiomyocyte; vEndocardial, ventricular endocardial; vFibro, ventricular fibroblast.

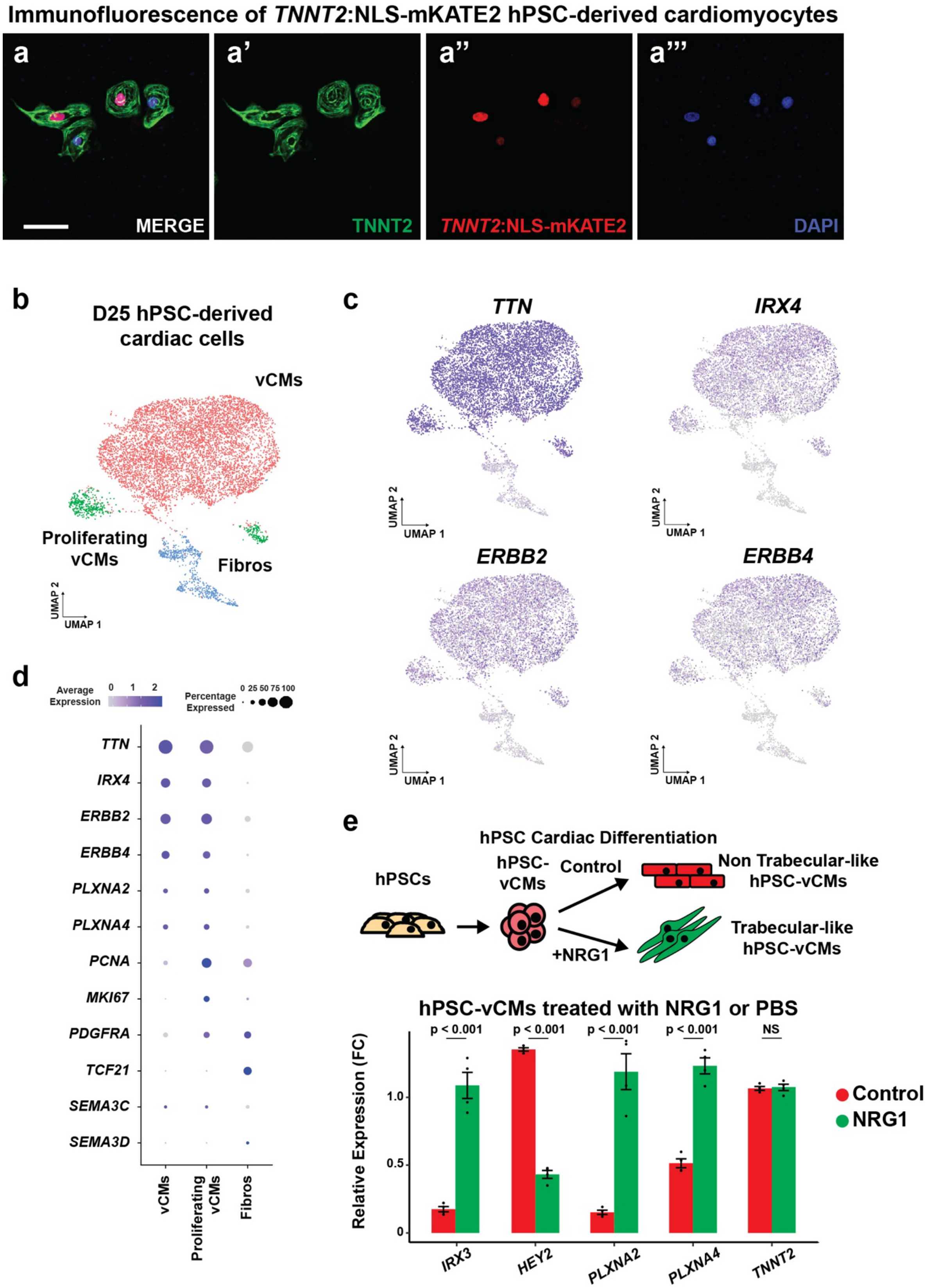
Immunofluorescence of TNNT2:NLS-mKATE2 hPSC-derived cardiomyocytes
a
a'
a''
a'''
MERGE
TNNT2
TNNT2:NLS-mKATE2
DAPI
b
D25 hPSC-derived cardiac cells
vCMs
Proliferating vCMs
Fibros
UMAP 2
UMAP 1
c
TTN
IRX4
ERBB2
ERBB4
d
Average Expression
0 1 2
Percentage Expressed
0 25 50 75 100
TTN
IRX4
ERBB2
ERBB4
PLXNA2
PLXNA4
PCNA
MKI67
PDGFRA
TCF21
SEMA3C
SEMA3D
vCMs
Proliferating vCMs
Fibros
e
hPSC Cardiac Differentiation
hPSCs
hPSC-vCMs
Control
+NRG1
Non Trabecular-like hPSC-vCMs
Trabecular-like hPSC-vCMs
hPSC-vCMs treated with NRG1 or PBS
p < 0.001
p < 0.001
p < 0.001
p < 0.001
NS
Relative Expression (FC)
1.0
0.5
0
IRX3
HEY2
PLXNA2
PLXNA4
TNNT2
Control
NRG1

Supplementary Figure 18. Human pluripotent stem cell (hPSC) cardiomyocyte differentiation protocol generates highly-enriched developing hPSC ventricular cardiomyocytes (hPSC-vCM), which differentiate into *PLXNA2*/*PLXNA4*+ trabecular-like vCMs after NRG1 treatment. a, Immunofluorescence analysis reveals that day 25 *TNNT2*:NLS-mKATE2 hPSC cardiomyocytes which express TNNT2 (**a'**) also express the NLS-mKATE2 fluorescent protein (**a''**). Images in **a'-a'''** are single channels from (**a**) merged image. Green - anti-TNNT2 immunostaining; red - *TNNT2*:NLS-mKATE2; and blue - DAPI. **b,** scRNA-seq performed on hPSC cardiomyocytes reveal three distinct cells (vCMs, Proliferating vCMs, and Fibros) as displayed by UMAP and colored by identified cell. **c**, UMAP of key marker genes related to vCMs shows that most hPSC derived cardiac cells are vCMs (*TTN*/*IRX4*+), and express the receptors required for receiving NRG1 (*ERBB2/ERRB4*). **d**, Marker gene analysis identifies cell clusters present at day 25 of hPSC cardiac differentiation. **e**, Schematic illustrates how hPSCs were differentiated into enriched ventricular cardiomyocytes, which were then treated with control PBS or NRG1 to create NLS-mKATE2+ non-trabecular and GFP+ trabecular-like hPSC vCMs, respectively. RT-qPCR shows that the expression of trabecular specific genes (*IRX3*, *PLXNA2*, and *PLXNA4*) increases when hPSC cardiomyocytes are treated with NRG1. N = 4 biologically independent samples per condition. Bar graphs represent average gene expression relative to the housekeeping gene TATA-box binding protein (*TBP*). Fibro, Fibroblast; hPSC, human pluripotent stem cell; vCM, ventricular cardiomyocyte. Error bars are SEM. *P* values by one-way ANOVA. NS, not significant. Scale bar, 50 µm.

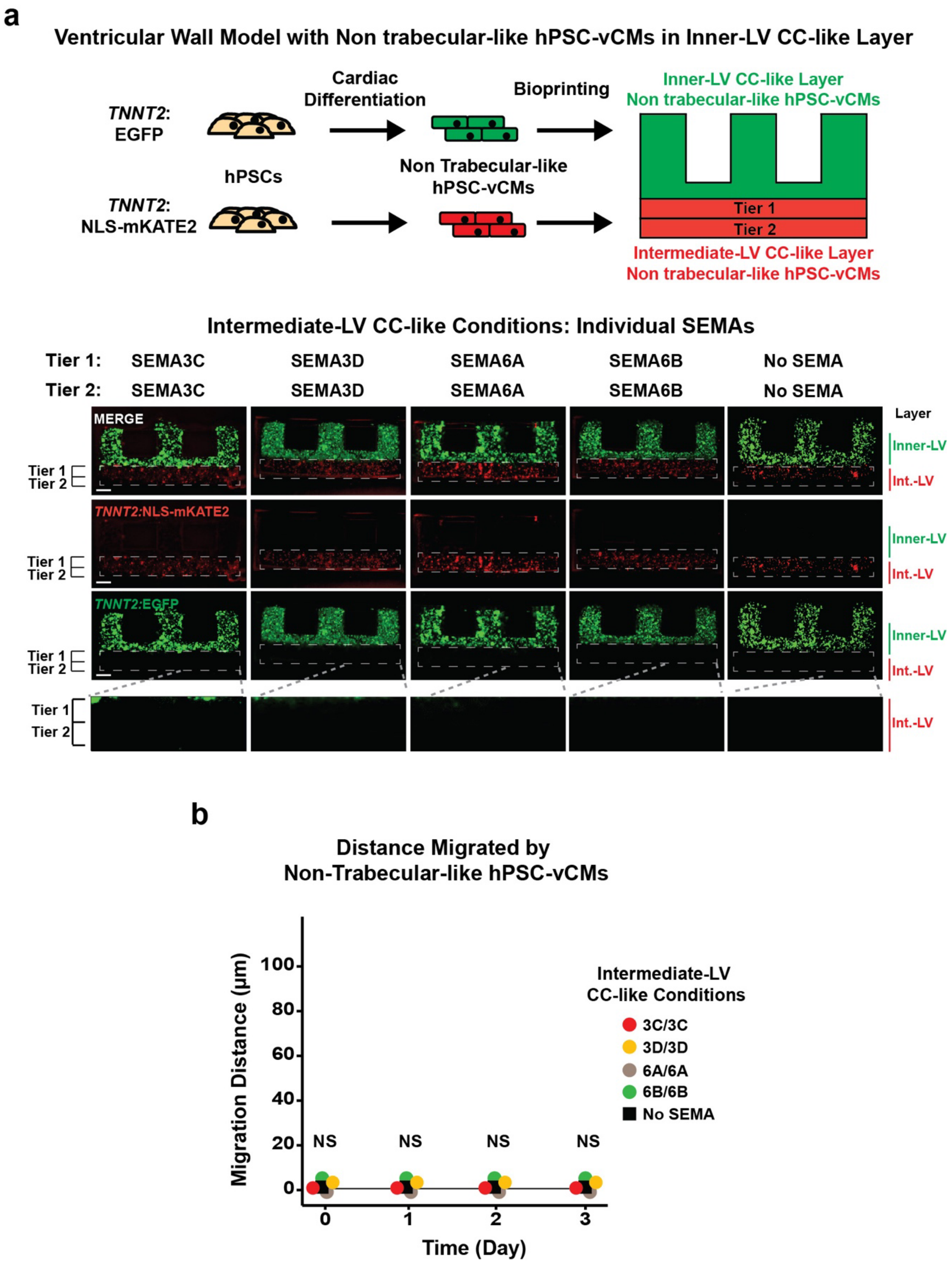
a
Ventricular Wall Model with Non trabecular-like hPSC-vCMs in Inner-LV CC-like Layer
Cardiac Differentiation
Bioprinting
TNNT2: EGFP
hPSCs
TNNT2: NLS-mKATE2
Non Trabecular-like hPSC-vCMs
Inner-LV CC-like Layer Non trabecular-like hPSC-vCMs
Tier 1
Tier 2
Intermediate-LV CC-like Layer Non trabecular-like hPSC-vCMs
Intermediate-LV CC-like Conditions: Individual SEMAs
Tier 1: SEMA3C SEMA3D SEMA6A SEMA6B No SEMA
Tier 2: SEMA3C SEMA3D SEMA6A SEMA6B No SEMA
Layer
MERGE
TNNT2:NLS-mKATE2
TNNT2:EGFP
Inner-LV
Int.-LV
b
Distance Migrated by Non-Trabecular-like hPSC-vCMs
Migration Distance (µm)
Time (Day)
Intermediate-LV CC-like Conditions
3C/3C
3D/3D
6A/6A
6B/6B
No SEMA
NS

Supplementary Figure 19. SEMA3C/3D and SEMA6A/6B do not affect the migration of non trabecular-like hPSC-vCMs. a, GFP+ non trabecular-like hPSC-vCMs do not migrate out of the inner-LV CC-like layer in response to any semaphorin. **b**, GFP+ non trabecular-like hPSC-vCM migration was measured under the different conditions tested. N = 3 per condition. White dashed lines in **a** outline the Intermediate-LV CC-like layer. CC, cellular community; hPSC-vCM, human pluripotent stem cell derived ventricular cardiomyocyte; Int, Intermediate; LV, left ventricle; NS, not significant. Scale bar, 100 μm.

Supplementary References

1. Asp, M. *et al.* A Spatiotemporal Organ-Wide Gene Expression and Cell Atlas of the Developing Human Heart. *Cell* **179**, 1647-1660.e19 (2019).
2. Litviňuková, M. *et al.* Cells of the adult human heart. *Nature* **588**, 466–472 (2020).
3. Tucker, N. R. *et al.* Transcriptional and Cellular Diversity of the Human Heart. *Circulation* **142**, 466–482 (2020).
4. Hocker, J. D. *et al.* Cardiac cell type-specific gene regulatory programs and disease risk association. *Sci. Adv.* **7**, (2021).
5. Cui, Y. *et al.* Single-Cell Transcriptome Analysis Maps the Developmental Track of the Human Heart. *Cell Rep.* **26**, 1934-1950.e5 (2019).
6. Yuen, M. *et al.* Leiomodin-dysfunction results in thin filament disorganization and nemaline myopathy. *J. Clin. Invest.* **124**, 4693–4708 (2014).
7. Whittington, H. J. *et al.* Over-expression of mitochondrial creatine kinase in the murine heart improves functional recovery and protects against injury following ischaemia–reperfusion. *Cardiovasc. Res.* **114**, 858–869 (2018).
8. Cao, J. *et al.* A human cell atlas of fetal gene expression. *Science* **370**, (2020).
9. Miao, Y. *et al.* Intrinsic Endocardial Defects Contribute to Hypoplastic Left Heart Syndrome. *Cell Stem Cell* **27**, 574-589.e8 (2020).
10. Ameen, M. *et al.* Integrative single-cell analysis of cardiogenesis identifies developmental trajectories and non-coding mutations in congenital heart disease. *Cell* **185**, 4937-4953.e23 (2022).
11. Li, G. *et al.* Transcriptomic Profiling Maps Anatomically Patterned Subpopulations among Single Embryonic Cardiac Cells. *Dev. Cell* **39**, 491–507 (2016).

12. Gladka, M. M. *et al.* Single-Cell Sequencing of the Healthy and Diseased Heart Reveals Cytoskeleton-Associated Protein 4 as a New Modulator of Fibroblasts Activation. *Circulation* **138**, 166–180 (2018).

13. Hill, M. C. *et al.* Integrated multi-omic characterization of congenital heart disease. *Nature* **608**, 181–191 (2022).

14. van Weerd, J. H. & Christoffels, V. M. The formation and function of the cardiac conduction system. *Development* **143**, 197–210 (2016).

15. Evans, S. M., Yelon, D., Conlon, F. L. & Kirby, M. L. Myocardial lineage development. *Circulation Research* **107**, 1428–1444 (2010).

16. Wessels, A. *et al.* Epicardially derived fibroblasts preferentially contribute to the parietal leaflets of the atrioventricular valves in the murine heart. *Dev. Biol.* **366**, 111–124 (2012).

17. Xie, L. *et al.* Tbx5-Hedgehog Molecular Networks Are Essential in the Second Heart Field for Atrial Septation. *Dev. Cell* **23**, 280–291 (2012).

18. Snarr, B. S., Wirrig, E. E., Phelps, A. L., Trusk, T. C. & Wessels, A. A spatiotemporal evaluation of the contribution of the dorsal mesenchymal protrusion to cardiac development. *Dev. Dyn.* **236**, 1287–1294 (2007).

19. Kanemaru, K. *et al.* Spatially resolved multiomics of human cardiac niches. *Nature* **619**, 801–810 (2023).

20. Korsunsky, I. *et al.* Fast, sensitive and accurate integration of single-cell data with Harmony. *Nat. Methods* **16**, 1289–1296 (2019).

21. Chaffin, M. *et al.* Single-nucleus profiling of human dilated and hypertrophic cardiomyopathy. *Nature* **608**, 174–180 (2022).

22. Sedmera, D., Pexieder, T., Vuillemin, M., Thompson, R. P. & Anderson, R. H. Developmental patterning of the myocardium. *Anat. Rec.* **258**, 319–337 (2000).

23. Wessels, A. & Sedmera, D. Developmental anatomy of the heart: A tale of mice and man. *Physiol. Genomics* **15**, 165–176 (2003).

24. Suto, F. *et al.* Plexin-A4 mediates axon-repulsive activities of both secreted and transmembrane semaphorins and plays roles in nerve fiber guidance. *J. Neurosci.* **25**, 3628–3637 (2005).

25. Toyofuku, T. *et al.* Repulsive and attractive semaphorins cooperate to direct the navigation of cardiac neural crest cells. *Dev. Biol.* **321**, 251–262 (2008).

26. Tawarayama, H., Yoshida, Y., Suto, F., Mitchell, K. J. & Fujisawa, H. Roles of semaphorin-6B and plexin-A2 in lamina-restricted projection of hippocampal mossy fibers. *J. Neurosci.* **30**, 7049–7060 (2010).

27. Chen, G. *et al.* Semaphorin-3A guides radial migration of cortical neurons during development. *Nat. Neurosci.* **11**, 36–44 (2008).

28. Ruediger, T. *et al.* Integration of Opposing Semaphorin Guidance Cues in Cortical Axons. *Cereb. Cortex* **23**, 604–614 (2013).

29. Houweling, A. C., Van Borren, M. M., Moorman, A. F. M. & Christoffels, V. M. Expression and regulation of the atrial natriuretic factor encoding gene Nppa during development and disease. *Cardiovascular Research* **67**, 583–593 (2005).

30. Bhavsar, P. K. *et al.* Developmental expression of troponin I isoforms in fetal human heart. *FEBS Lett.* **292**, 5–8 (1991).

31. Bao, Z. Z., Bruneau, B. G., Seidman, J. G., Seidman, C. E. & Cepko, C. L. Regulation of chamber-specific gene expression in the developing heart by Irx4.

Science **283**, 1161–1164 (1999).

32. Acharya, A. *et al.* The bHLH transcription factor Tcf21 is required for lineage-specific EMT of cardiac fibroblast progenitors. *Dev.* **139**, 2139–2149 (2012).
