# Supplementary material for: Spatially organized cellular communities form the developing human heart
Source: Nature. 2024 Mar 13;627(8005):854–64. doi: 10.1038/s41586-024-07171-z (PMC10972757; doi:10.1038/s41586-024-07171-z)
Supplement: Supplementary file 2 — Reporting Summary [file 41586_2024_7171_MOESM2_ESM.pdf]

nature portfolio

Corresponding author(s): Quan Zhu
Neil C. Chi

Last updated by author(s): Jan 18, 2024

Reporting Summary

Nature Portfolio wishes to improve the reproducibility of the work that we publish. This form provides structure for consistency and transparency in reporting. For further information on Nature Portfolio policies, see our Editorial Policies and the Editorial Policy Checklist.

Statistics

For all statistical analyses, confirm that the following items are present in the figure legend, table legend, main text, or Methods section.

n/a	Confirmed	
☐	☒	The exact sample size (*n*) for each experimental group/condition, given as a discrete number and unit of measurement
☐	☒	A statement on whether measurements were taken from distinct samples or whether the same sample was measured repeatedly
☐	☒	The statistical test(s) used AND whether they are one- or two-sided<br>*Only common tests should be described solely by name; describe more complex techniques in the Methods section.*
☐	☒	A description of all covariates tested
☐	☒	A description of any assumptions or corrections, such as tests of normality and adjustment for multiple comparisons
☐	☒	A full description of the statistical parameters including central tendency (e.g. means) or other basic estimates (e.g. regression coefficient) AND variation (e.g. standard deviation) or associated estimates of uncertainty (e.g. confidence intervals)
☐	☒	For null hypothesis testing, the test statistic (e.g. *F*, *t*, *r*) with confidence intervals, effect sizes, degrees of freedom and *P* value noted<br>*Give P values as exact values whenever suitable.*
☒	☐	For Bayesian analysis, information on the choice of priors and Markov chain Monte Carlo settings
☒	☐	For hierarchical and complex designs, identification of the appropriate level for tests and full reporting of outcomes
☒	☐	Estimates of effect sizes (e.g. Cohen's *d*, Pearson's *r*), indicating how they were calculated

Our web collection on statistics for biologists contains articles on many of the points above.

Software and code

Policy information about availability of computer code

Data collection	Fastqs collected from 10X Genomics Chromium Single Cell 3' Expression kit were mapped with Cell Ranger v3.0.1 (10X Genomics). MERFISH imaging data was collected using custom Python (v3.9) code to control the microscope (available here from the Zhuang Lab: https://github.com/ ZhuangLab). Quantitative Real-Time PCR data was collected with CFX Manager v3.1. Data from mouse heart sections were collected with NDP View 2 software (Hamamatsu) and QuPath v0.4.3.
Data analysis	The pipeline for generating the encoding probes used in the MERFISH studies is available from: https://github.com/bil022/ProbeDesign. The pipeline for processing the MERFISH dataset including cell segmentation and assigning barcodes to cells is available from: https://github.com/epigen-UCSD/merfish_tools. Custom code used for analyzing the scRNA-seq and MERFISH datasets in this study is available from: https://github.com/ChiLab-UCSD/Heart_MERFISH_analysis. Other packages used in data analysis include: Cellpose (v1.0.2); Seurat (v4.0.1); DoubletFinder (v2.0); SCCAF (v0.0.10); scArches (v0.5.9); scVI (v1.0.3); pySCENIC (v0.12.1); CellChat (v1.6.1); Waddington-OT (v1.0.8); MERlin (v0.6.1); Scanpy (v1.8); scikit-learn (v0.22); python (v3.9); R (v4.2.0)

For manuscripts utilizing custom algorithms or software that are central to the research but not yet described in published literature, software must be made available to editors and reviewers. We strongly encourage code deposition in a community repository (e.g. GitHub). See the Nature Portfolio guidelines for submitting code & software for further information.

Data

Policy information about availability of data

All manuscripts must include a data availability statement. This statement should provide the following information, where applicable:

- Accession codes, unique identifiers, or web links for publicly available datasets
- A description of any restrictions on data availability
- For clinical datasets or third party data, please ensure that the statement adheres to our policy

Data availability statement is included in the manuscript, which states:
Raw sequencing data for the in vivo studies is available from dbGAP under accession number (phs002031). Raw sequencing data for the in vitro studies is available from CIRM CESCG (https://cirm.ucsc.edu) under accession number (chiCardiomyocyte1). Processed scRNA-seq data is accessible on the UCSC Cell Browser (https://cells.ucsc.edu/?ds=hoc). MERFISH imaging data is available through Dryad (doi:10.5061/dryad.w0vt4b8vp). The human reference genome sequences (hg38) can be downloaded from ncbi_refseq: https://hgdownload.soe.ucsc.edu/goldenPath/hg38/bigZips/genes/.

Human research participants

Policy information about studies involving human research participants and Sex and Gender in Research.

Reporting on sex and gender	Sex and gender were not considered in the study design and were not collected.
Population characteristics	Eleven hearts between the ages of 9 to 16 post conception weeks. Age was considered as a covariate-relevant population characteristic.
Recruitment	The heart samples were collected by the University of California, San Diego (UCSD) Perinatal Biorepository's Developmental Biology Resource (DBR). All donors gave informed consent for the collection of these tissues by medical termination. Age of the sample was measured using the crown rump length (CRL) method, and all samples were screened for and found to be absent of structural fetal abnormalities. Tissue samples were collected and transported in buffer containing 10 mM HEPES pH 7.8, 130 mM NaCl, 5 mM KCl, 10 mM Glucose, 10 mM 2,3-Butanedione monoxime (BDM), 10 mM Taurine, 1 mM EDTA, and 0.5 mM NaH2PO4, and overall morphology was checked under a stereotaxic dissection microscope (Leica).
Ethics oversight	Heart samples were collected in strict observance of the legal and institutional ethical regulations. The heart samples were collected under a University of California, San Diego (UCSD) Human Research Protections Program Committee Institutional Review Board (IRB)-approved protocol (IRB #081510) by the UCSD Perinatal Biorepository's Developmental Biology Resource (DBR), and all experiments were performed within the guidelines and regulations set forth by the IRB (IRB #101021, registered with the DBR). Ethical requirements for data privacy include that sequence-level data (e.g. fastq files) be shared through controlled-access databases.

Note that full information on the approval of the study protocol must also be provided in the manuscript.

Field-specific reporting

Please select the one below that is the best fit for your research. If you are not sure, read the appropriate sections before making your selection.

☒ Life sciences ☐ Behavioural & social sciences ☐ Ecological, evolutionary & environmental sciences

For a reference copy of the document with all sections, see nature.com/documents/nr-reporting-summary-flat.pdf

Life sciences study design

All studies must disclose on these points even when the disclosure is negative.

Sample size	Sample size of at least two was chosen to provide sufficient material for scRNA-seq assays, and ensure replication of the results with affordable cost. For MERFISH, three replicate sections from a 13 post conception week heart and one section of 15 post conception week ventricles were imaged for MERFISH, providing a total of ~280,000 cells which was provided a sufficient number of single-cell profiles and gave sufficient statistics for the effect sizes of interest. All other experiments, including hPSC and mouse experiments, have at least three independent biological replicates which gave sufficient statistics for the effect sizes of interest. Sample sizes were not predetermined utilizing statistical methods and sample size was determined empirically.
Data exclusions	No data was excluded.
Replication	Reported scRNA-seq results were replicated from two biological replicates for each stage of development. Reported MERFISH results were replicated using three biological sections from one 13 post conception week heart, and reported ventricle results from additional 15 post conception week ventricles, and correlation analyses were conducted to ensure the consistency between the replicates. Reported mouse results were replicated from three animals under each condition. All attempts at replication were successful.
Randomization	Data for scRNA-seq and MERFISH was collected from all available samples and no randomization was necessary. For the studies utilizing

Randomization	human pluripotent stem cell lines, treatment with NRG1 was randomly assigned. For the animal studies, animals were randomly chosen from each genotype and timepoint.
Blinding	The investigators were not blinded during collection as no subjective measurements were taken. Blinding during analysis was not necessary as all of the results were analyzed with the use of unbiased analysis and software tools that are not affected by the sample.

Reporting for specific materials, systems and methods

We require information from authors about some types of materials, experimental systems and methods used in many studies. Here, indicate whether each material, system or method listed is relevant to your study. If you are not sure if a list item applies to your research, read the appropriate section before selecting a response.

Materials & experimental systems

n/a	Involved in the study	
☐	☒	Antibodies
☐	☒	Eukaryotic cell lines
☒	☐	Palaeontology and archaeology
☐	☒	Animals and other organisms
☒	☐	Clinical data
☒	☐	Dual use research of concern

Methods

n/a	Involved in the study	
☒	☐	ChIP-seq
☐	☒	Flow cytometry
☒	☐	MRI-based neuroimaging

Antibodies

Antibodies used	Antibody: Alexa Fluor® 647 Mouse Anti-Cardiac Troponin T<br>Supplier: BD Biosciences<br>Cat No: 565744<br>Clone: 13-11 (RUO)
Validation	Alexa Fluor® 647 Mouse Anti-Cardiac Troponin T: https://www.bdbiosciences.com/en-eu/products/reagents/flow-cytometry-reagents/research-reagents/single-color-antibodies-ruo/alexa-fluor-647-mouse-anti-cardiac-troponin-t.565744

Eukaryotic cell lines

Policy information about cell lines and Sex and Gender in Research

Cell line source(s)	H9-hTnnTZ-pGZ-D2 human pluripotent stem cell (hPSC) line was purchased from WiCell. An additional TNNT2:NLS-mKATE2-T2A-BsdR RUES2 hPSC cardiomyocyte reporter line was generated that specifically expresses the mKATE2 fluorescent protein containing a nuclear localization signal (NLS-mKATE2) in differentiated cardiomyocytes, as detailed in the methods.
Authentication	H9-hTnnTZ-pGZ-D2 and TNNT2:NLS-mKATE2-T2A-BsdR RUES2 hPSC reporter transgenic lines were authenticated with Short Tandem Repeat (STR) profiling analysis and immunofluorescence.
Mycoplasma contamination	Cell lines tested negative for mycoplasma contamination by PCR
Commonly misidentified lines<br>(See ICLAC register)	None used in this study

Animals and other research organisms

Policy information about studies involving animals; ARRIVE guidelines recommended for reporting animal research, and Sex and Gender in Research

Laboratory animals	The information of Tcf21-CreERT2; Sema3c fl/fl mice are included in the manuscript, within the methods.<br>All animals used for timed matings were aged 8-10 weeks (female) or 8-10 weeks (male) of age. E12.5, E14.5, E17.5, and P1 mouse embryos were collected for histological analysis. Mice were housed on a 12 hour light/dark cycle (6am-6pm light cycle), with a temperature between 20-22 degrees Celsius, and a humidity range of 30-70%.
Wild animals	The study did not involve wild animals.
Reporting on sex	Both male and female embryos were used in this study; the embryos were not genotyped to determine the sex.
Field-collected samples	The study did not involve samples collected from the field.
Ethics oversight	All protocols concerning animal use were approved by the Institutional Animal Care and Use Committee (IACUC) at UCSD and were accredited by the Association for Assessment and Accreditation of Laboratory Animal Care (AAALAC).

Note that full information on the approval of the study protocol must also be provided in the manuscript.

Flow Cytometry

Plots

Confirm that:

☒ The axis labels state the marker and fluorochrome used (e.g. CD4-FITC).

☒ The axis scales are clearly visible. Include numbers along axes only for bottom left plot of group (a 'group' is an analysis of identical markers).

☒ All plots are contour plots with outliers or pseudocolor plots.

☒ A numerical value for number of cells or percentage (with statistics) is provided.

Methodology

Sample preparation	As described in the Methods section. Briefly, the single cells were dissociated and were resuspended in PBS supplemented with 5% FBS and sorted on a Sony SH800 sorter.
Instrument	Sony SH800 sorter
Software	Proprietary Sony SH800 Software and FlowJo (v10)
Cell population abundance	Not applicable because we sorted as many live single cells as necessary to complete downstream scRNA-seq processing
Gating strategy	Single cells were gated based on SSC and BSC. Live cells were gated based on DAPI (DAPI negative).

☒ Tick this box to confirm that a figure exemplifying the gating strategy is provided in the Supplementary Information.
